# A Systematic Review and an Updated Meta-Analysis of Fenestrated/Branched Endovascular Aortic Repair of Chronic Post-Dissection Thoracoabdominal Aortic Aneurysms

**DOI:** 10.3390/jcm13020410

**Published:** 2024-01-11

**Authors:** Spyridon N. Mylonas, Tuna Aras, Bernhard Dorweiler

**Affiliations:** Department of Vascular and Endovascular Surgery, Faculty of Medicine and University Hospital of Cologne, University of Cologne, 50937 Cologne, Germany; tuna.aras@uk-koeln.de (T.A.); bernhard.dorweiler@uk-koeln.de (B.D.)

**Keywords:** aortic dissection, stent graft, fenestrated, physician-modified, branched, mortality

## Abstract

The objective of this study is to present the current outcomes of fenestrated/branched endovascular repair (F/BEVAR) for post-dissection thoracoabdominal aortic aneurysms (PDTAAAs). A systematic review of the literature according to PRISMA guidelines up to October 2023 was conducted (protocol CRD42023473403). Studies were included if ≥10 patients were reported and at least one of the major outcomes was stated. A total of 10 studies with 585 patients overall were included. The pooled estimate for technical success was 94.3% (95% CI 91.4% to 96.2%). Permanent paraplegia developed with a pooled rate of 2.5% (95% CI 1.5% to 4.3%), whereas a cerebrovascular event developed with a pooled rate of 1.6% (95% CI 0.8% to 3.0%). An acute renal function impairment requiring new-onset dialysis occurred with a pooled rate of 2.0% (95% CI 1.0% to 3.8%). Postoperative respiratory failure was observed with a pooled estimate of 5.5% (95% CI 3.8% to 8.1%). The pooled estimate for 12-month overall survival was 90% (95% CI 85% to 93.5%), and the pooled estimates for 24-month and 36-month survival were 87.8% (95% CI 80.9% to 92.5%) and 85.5% (95% CI 76.5% to 91.5%), respectively. Freedom from reintervention was estimated at 83.9% (95% CI 75.9% to 89.6%) for 12 months, 82.8% (95% CI 68.7% to 91.4%) for 24 months and 76.1% (95% CI 60.6% to 86.8%) for 36 months. According to the present findings, F/BEVAR can be performed in PD-TAAAs with high rates of technical success and good mid-term results.

## 1. Introduction

Aortic dissection is a progressive entity with a gradual transition from a subacute to a chronic state. Although any clear distinction may be arbitrary, beyond the subacute phase (>14 days–90 days from onset), there is, by definition, a chronic scenario [1,2]. It is estimated that a proportion of patients, ranging from 20% to 40%, surviving an acute aortic dissection will develop aortic-related complications, which may require further treatment [3,4,5,6,7]. Aneurysmal dilatation and rapid growth of aneurysms are the most frequent indications for the treatment of chronic aortic dissection [8,9,10]. In addition, given the lower plasticity of the dissection flap in the chronic phase, -which limits the resulting aortic remodeling that can be achieved by covering the primary entry tear- the dissected segment of the aorta at the distal end of the stent graft (i.e., thoracoabdominal aorta) remains untreated and may dilate over time [11,12,13]. Several studies have demonstrated a risk of rupture of almost 20% with an aortic diameter between 50 and 60 mm [14,15]. Therefore, the recent guidelines recommend that an aortic diameter greater than 60 mm should be considered as an indication for treatment in patients at reasonable surgical risk with chronic aortic dissection and thoracoabdominal extension. In patients with an aortic diameter of 56–59 mm, treatment may be considered [1,16,17].

Patients with aneurysms precipitated by chronic dissection are different from those with degenerative atherosclerotic aneurysms: they are typically younger, have fewer comorbidities and are more likely to have heritable aortic disease [16]. Thus, open repair was considered the only therapeutic option a few decades ago. However, despite the improvements in organ and spinal cord protection strategies, it is still associated with considerable mortality and morbidity rates, even in centers of excellence [18,19,20,21].

On the other hand, fenestrated and branched EVAR (F/BEVAR), which emerged as new techniques to treat more complicated cases with an endovascular approach, are being used with increasing frequency for the treatment of post-dissection thoracoabdominal aneurysms (PDTAAAs) [22,23,24]. However, early experiences with F/BEVAR for PDTAAAs have underlined specific difficulties due to a noncompliant dissection septum and compromised true lumen, variable (from different lumens) origins of the visceral vessels and the common use of diseased aortic landing zones [24].

The objective of this study is to present the current outcomes of F/BEVAR for PDTAAAs. A systematic review of the currently published literature on FEVAR/BEVAR for chronic post-dissection thoracoabdominal aortic aneurysms is undertaken, and the eligible studies are combined into a meta-analysis with the intention of evaluating the safety, efficacy and durability of this treatment option.

## 2. Material and Methods

### 2.1. Study Protocol and Registration

The Preferred Reporting Items for Systematic Reviews and Meta-analyses (PRISMA) and the Meta-analyses of Observational Studies in Epidemiology (MOOSE) guidelines were applied for the design, conduction and reporting of this meta-analysis [25,26]. In addition, the present meta-analysis has been registered in PROSPERO public database prior to study initiation (CRD42023473403).

### 2.2. Eligibility Criteria

A study was considered eligible for the present meta-analysis if:

- It reported on the application of F/BEVAR for treatment of PDTAAs. At least one target vessel (including renal arteries, coeliac trunk, or mesenteric arteries) had to be included with either a branched (including side branch, inner branch, antegrade and retrograde) or fenestrated device (custom-made, off-the-shelf, physician-modified).
- It included ≥10 patients. With the intention of averting the bias of learning curve, small case series and case reports were excluded.
- At least one of the major outcomes (primary technical success, postoperative complications, mortality, reintervention rate) was stated.

Articles in languages other than English were excluded. Animal studies were not included. Reports on PDTAAA repair by conventional surgical or hybrid approaches were also excluded. Moreover, reports on isolated chronic post-dissection arch aneurysms were also not included in the present meta-analysis.

Furthermore, several studies included patients with PDTAAAs as a subset of the entire study cohort. These were included in the present review if separate data for this patient subgroup were provided. When multiple publications on the same patient population were identified or study populations overlapped, only the latest report was included unless the reported outcomes were mutually exclusive.

### 2.3. Search Strategy

A multiple electronic search up to October 2023 was performed in Medline (database provider PubMed), Web of Science Core Collection, EMBASE (database provider Ovid) and Cochrane Central Register of Controlled Trials databases for articles reporting on FEVAR/BEVAR for chronic post-dissection thoracoabdominal aneurysms. These databases were searched with an unrestricted search strategy using exploded medical subject heading (MeSH) terms, including “chronic aortic dissection”, “dissecting aneurysm”, “aortic aneurysm”, “endovascular repair”, “fenestrated”, “branched”, “physician modified stent-graft”, “surgeon modified stent-graft”, “thoracoabdominal aneurysm” or combination of them. All studies were independently assessed by two reviewers (S.M. and T.A.) at the title and abstract levels, and the full texts of the studies were retrieved. Disagreements were resolved through consensus. In addition, a “snowball process” was conducted in the reference lists of the investigated articles to capture additional eligible articles.

### 2.4. Data Extraction Process

Data were independently extracted by two reviewers (S.M. and T.A.) and collected into a pre-designed data extraction form. The following data were extracted: first author’s name; center; publication year; study design; recruitment period; total number of patients; participants’ demographics and baseline characteristics; maximal PDTAAA diameter; design of stent grafts used (custom-made, off-the-shelf, physician-modified); setting of the procedure (rupture, symptomatic, large diameter or rapid growth or elective); and intraoperative details (adjunctive procedures, number of target vessels and their origin, need of perforation of the dissection membrane). Primary outcome data (efficacy) included technical success, defined as successful deployment of the main aortic graft, successful catheterization of all fenestration(s)/branches and deployment of the intended bridging stents/stent grafts into the target vessel(s), with patency of the endograft and all target vessels without any EL type I or III as evidenced by intra-operative completion angiography [27]. Further outcome variables (safety) included in-hospital or 30-day mortality, postoperative neurological complications (stroke or transient ischemic attack; paraplegia or paraparesis), myocardial infarction, respiratory failure requiring prolonged ventilation (>24 h or re-intubation), renal failure requiring dialysis and bowel requiring surgery. Durability outcome data included follow-up period, late complications and reinterventions, endoleak detection during follow-up, survival, target vessel patency and freedom of reintervention. In case of discrepancies in obtained results, the relative articles were reanalyzed by the 2 reviewers, and consensus was reached.

### 2.5. Quality Assessment of the Eligible Studies

Methodological quality and robustness of the results of the eligible articles were assessed according to JBI’s critical appraisal tool evaluating the following domains: definition of inclusion criteria, description of study population, reporting of interventional parameters, reporting of outcomes or follow-up results and statistical analysis applied [28] (Supplementary Table S1).

### 2.6. Statistical Analysis

Standard descriptive statistics (reported as mean with 95% confidence interval (CI)) were used to summarize demographical and baseline data of the recruited patients from all eligible studies. Furthermore, separate meta-analyses were carried out on all included studies for technical success and neurological complications (stroke, TIA, paraplegia), as well as 30-day/in-hospital mortality, survival and freedom of reintervention rates. For events during follow-up (late reinterventions, late endoleaks), we calculated the incidence rates (IRs) with 95% confidence intervals (95% CIs) per 100 patient-years (p-ys) as the number of patients with outcome events occurring during the specific time period divided by the total number of p-ys. In contrast to crude percentages, IRs take into account differences in the follow-up duration among the eligible studies. The corresponding crude percentages and IRs (95% confidence intervals) were, thereafter, transformed into quantities using the Freeman–Tukey variant of the arcsine square-root-transformed proportion [29]. The pooled effect estimates were calculated as the back-transformation of the weighted mean of the transformed proportions, using inverse arcsine variance weights for the fixed effects model and DerSimonian–Laird weights for the random effects model [30]. Heterogeneity among studies was estimated by chi-squared test, and Cochran Q score (reported as I^2^) and corresponding Egger’s regression tests were used as a measure of estimating publication bias [31]. Sensitivity analysis was performed regarding the design of the used stent grafts (manufactured or physician-modified). The meta-analysis was conducted by using the Comprehensive Meta-analysis Package V4 (Biostat, Englewood, NJ, USA) statistical software.

## 3. Results

**Figure 1:**
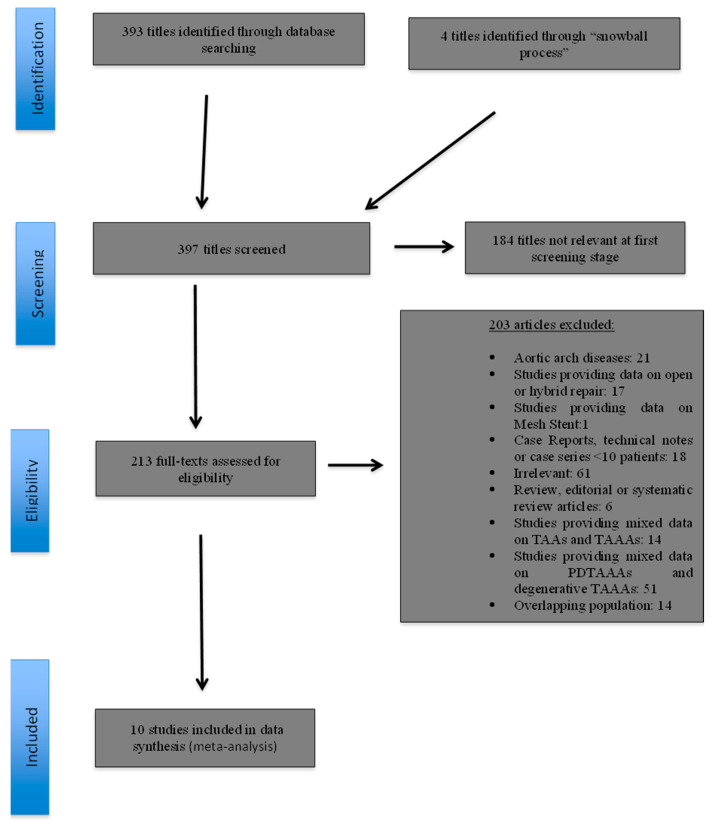
Study flow chart (“Preferred Reporting Items for Systematic reviews and Meta-Analysis” diagram).

The initial electronic research yielded a total of 393 study titles; four further studies were identified through the “snowball” process. The review of the titles and abstracts revealed that 184 studies were irrelevant at the first screening stage. Thus, 213 reports were evaluated further. Of these, 203 were excluded for one or more of the following reasons: provided data on aortic arch disease (*n* = 21); reported on open or hybrid repair (*n* = 17); provided data on Mesh Stent (*n* = 1); case reports, technical notes or case series with >10 patients (*n* = 18); editorial and review articles (*n* = 6); provided mixed data on TAAs and TAAAs (*n* = 14); provided mixed data on degenerative TAAAs and PDTAAAs (*n* = 51); were irrelevant (*n* = 61); and/or reported on overlapping patient populations (*n* = 14) (Figure 1). Finally, 10 studies [32,33,34,35,36,37,38,39,40,41], with a total of 585 patients (1255.65 p-ys), were deemed eligible for inclusion in the meta-analysis (Table 1) (Figure 1).

Among the 585 patients included in our analysis, 456 (77.9%) were men, and the mean age was 61.6 years (95 CI 58.5 to 64.7 years). Patient demographics and comorbidities are provided in Table 2. The mean maximum aneurysm diameter was 60.2 mm (95% CI 56.4 to 63.9). Only five studies [34,35,36,38,40] provided data regarding the nature of dissection for a total of 223 patients; 65 of them (29.1%) had a residual type A AD, and 158 (70.9%) had a chronic type B AD. The vast majority of the patients (78.4%) were operated on in an elective setting, whereas 23.1% and 2.9% of the patients were operated on in an urgent or emergency setting, respectively. A variety of fenestrated or branched stent grafts were used: 378 patients received a custom-made stent graft, 162 patients were treated with a physician-modified stent graft (PMSG) and 45 patients were treated with off-the-shelf branched devices (Cook T-Branch, Cook Inc., Bloomington, IN, USA).

Regarding target vessels (TVs), data were provided in eight studies [32,33,34,35,37,38,39,40], with a total of 1815 vessels being incorporated in the repairs with either a fenestration, branch or a scallop. Four studies [33,34,35,40] describing 143 patients with 521 TVs reported a rate of need for perforation of the dissection flap of 3.6% (19/521). Details on TV origin and the modality of TV incorporation are provided in Supplementary Table S2.

### 3.1. Outcomes

Efficacy: The pooled estimate for technical success was 94.3% (95% CI 91.4% to 96.2%, I^2^ = 11%) (Supplementary Figure S1). Reasons for not achieving technical success included failure to complete catheterization/stent deployment in 15 patients and type I or III endoleak in 8 patients. In one patient treated with a PMSG, CT and SMA were inadvertently cannulated through the same limb and CT was sacrificed [33]. In three further patients, the reason for technical failure was not described [35,37].

Safety: The mean length of ICU stay was 2.9 days (95% CI 0.3 to 6.2 days), and the mean length of hospital stay was 10.7 days (95% CI 8.6 to 12.7 days). The pooled estimate for the 30-day/in-hospital mortality was 2.7% (95% CI 1.6% to 4.4%, I^2^ = 0%) (Supplementary Figure S2). Permanent paraplegia developed with a pooled rate of 2.5% (95% CI 1.5% to 4.3%, I^2^ = 0%) (Supplementary Figure S3), whereas a cerebrovascular event (stroke/TIA) developed with a pooled rate of 1.6% (95% CI 0.8% to 3.0%, I^2^ = 0%) (Supplementary Figure S4). Postoperative respiratory failure requiring prolonged mechanical ventilation or reintubation was observed with a pooled estimate of 5.5% (95% CI 3.8% to 8.1%, I^2^ = 3.4%) (Supplementary Figure S5), whereas mesenteric ischemia requiring laparotomy was found with a pooled rate of 1.3% (95% CI 0.6% to 2.8%, I^2^ = 0%) (Supplementary Figure S6). An acute renal function impairment requiring new-onset dialysis and a myocardial infarction occurred with pooled rates of 2.0% (95% CI 1.0% to 3.8%, I^2^ = 0%) (Supplementary Figure S7) and 1.5% (95% CI 0.7% to 3.0%, I^2^ = 0%) (Supplementary Figure S8), respectively.

Durability: The pooled estimate for 12-month overall survival was 90% (95% CI 85% to 93.5%, I^2^ = 5.2%) (Supplementary Figure S9). Among seven studies [32,35,36,38,39,40,41] providing data, the pooled estimates for 24-month and 36-month survival were 87.8% (95% CI 80.9% to 92.5%, I^2^ = 54%) and 85.5% (95% CI 76.5% to 91.5%, I^2^ = 68%), respectively (Supplementary Figures S10 and S11).

TV patency data were provided in eight studies [33,34,35,36,37,38,39,40] for 12 months with a pooled estimate of 97.6% (95% CI 94.9% to 98.8%, I^2^ = 0%) (Supplementary Figure S12) and in five studies [35,36,38,39,40] for 24 months with a pooled rate of 96.7% (95% CI 93.5% to 98.3%, I^2^ = 0%) (Supplementary Figure S13). The 36-month TV patency was estimated at 94.3% (95% CI 91.8% to 96.1%, I^2^ = 0%) (Supplementary Figure S14) among six eligible studies [32,35,36,38,39,40].

Freedom from reintervention was estimated at 83.9% (95% CI 75.9% to 89.6%, I^2^ = 71%) for 12 months [32,33,34,35,36,37,38,39,40], 82.8% (95% CI 68.7% to 91.4%, I^2^ = 87.8%) for 24 months [32,35,36,38,39,40] and 76.1% (95% CI 60.6% to 86.8%, I^2^ = 87.4%) for 36 months [32,35,36,39,40] (Supplementary Figures S15–S17).

The pooled reintervention IR per 100 p-ys was 12.6 (95% CI 7.3 to 17.9, I^2^ = 95%) (Supplementary Figure S18), whereas the pooled IR per 100 p-ys for type I/III endoleak was 5.5 (95% CI 2.3 to 8.7, I^2^ = 85.7%) (Supplementary Figure S19).

Data regarding the aneurysm sac behavior after treatment were available for only 350 patients. Among them, in 148 patients (42.3%), a sac regression of at least 5 mm was observed; in 149 patients (42.6%), the sac remained stable; and an increase of more than 5 mm was observed in 53 patients (15%).

### 3.2. Sensitivity Analysis

Sensitivity analysis was performed for technical success rate, 30-day/in-hospital mortality rate, 12-month survival, 12-month TV patency rate and 12-month freedom from reintervention rate. Overall, a tendency toward better outcomes was found if studies with CMD and/or off-the-shelf stent grafts were excluded. The results of the sensitivity analysis are detailed in Table 3.

## 4. Discussion

This meta-analysis provides the most contemporary insight into the application of F/BEVAR for the treatment of PDTAAAs. According to our findings, the method demonstrated favorable results regarding efficacy (technical success rate of 94.3%), safety (30-day/in-hospital mortality rate of 2.7%, postoperative permanent paraplegia rate of 2.5%, new-onset dialysis rate of 2.0%) and mid-term durability (overall survival and freedom from reintervention of 90%, 87.8% and 85.5% and 83.9%, 82.8% and 76.1% after 12, 24 and 36 months, respectively).

However, endovascular management of PDTAAAs is associated with specific anatomic and technical challenges; achieving adequate and healthy landing zones often requires extensive aortic coverage; and vessel catheterization can be difficult because of true lumen compression, separate origins from true or false lumen or extension of the dissection flap into the renal and mesenteric vessels [24,42,43]. Staging of the procedure and technological advancements, such as low-profile stent grafts, tapered device diameter and double-reducing ties, as well as pre-canulated fenestrations or inner branches, have been proven to be helpful in overcoming these challenges [32,34,37]. An excellent technical success rate of 94.3% was found in this meta-analysis.

Moreover, several techniques have been suggested to fenestrate the dissection flap in order to gain access to target vessels originating from the false lumen. These include the use of a stiff wire with a steerable sheath, a transjugular intrahepatic portosystemic shunt needle (TIPS needle) or a wire connected to electrocautery [43,44,45]. It should, however, be mentioned that target vessels with origins from false lumen usually have adjacent re-entrances, and these techniques are rarely needed. In our study, perforation of the dissection flap was only performed in 3.6% of the target vessels.

A major parameter determining the technical success and short- and long-term results of F/BEVAR technology is the incorporation of TVs in the repair. Among the eligible studies were 15 patients with technical failure due to an inability to complete catheterization/stent deployment in TVs. The pooled estimates for TV patency were 97.6%, 96.7 and 94.3% at 12, 24 and 36 months, respectively, which are comparable to those reported for degenerative aneurysms [46,47]. Moreover, it has been observed that renal mating stents perform worse in comparison to those for the coeliac trunk or superior mesenteric artery, and fenestrations perform better when compared to branches in the mid- and long term [47,48]. The renal artery movement during the respiratory cycle, which usually occurs at a 15 mm distance from the renal artery ostium, may contribute to mating stent instability or even occlusion. In the case of PDTAAAs with dissected TVs, the mating stent should usually be longer and reach a deeper landing, compromising the patency rates. Similar to other series on degenerative TAAAs, the majority of TV occlusions referred to renal arteries.

Another major concern in the endovascular treatment of thoracoabdominal aorta, especially for PDTAAAs, is the risk of postoperative complications. The devastating complication of SCI remains a great threat. However, it must be noted that the incidence of permanent SCI was homogeneously low (2.5%) among eligible studies, underlining the high awareness regarding this complication and the wide adaption of suggested efforts to prevent this, though various protocols regarding CSF drainage were implemented. Furthermore, acute renal function impairment requiring new-onset dialysis was observed with a pooled rate of 2.0%, which is comparable to the reported rates after open repair [49]. On the contrary, postoperative respiratory failure was found with a pooled incidence of 5.5%, which is not neglectable, but it is clearly lower risk compared to open repair [49].

The high rates of secondary aortic reinterventions also affect the durability of F/BEVAR for PDTAAAs and raise some concerns. We found a pooled estimation for freedom from reintervention of 83.9%, 82.8% and 76.1% after 12, 24 and 36 months, respectively. The most common reason for reintervention was type I/III endoleak, with a pooled IR per 100 p-ys of 5.5. This explains the observation of other authors that reinterventions after F/BEVAR for PDTAAAs occur shortly after the initial procedure, while patients with degenerative TAAAs have a more gradual rate of reinterventions [34]. Moreover, the great heterogeneity observed among studies can be attributed to the variety of adjunct procedures performed during the index procedure. In a study by Wang et al. [38] on 39 patients with PDTAAAs treated with PMSGs, almost half of the patients had adjunctive procedures (e.g., false lumen embolization) during the index procedure; this may explain the low rates of secondary procedures over time. However, regardless of adjunct procedures or reinterventions, more than 80% of the patients analyzed in this meta-analysis showed a shrinkage or a stability of the aneurysm sac.

Another interesting finding shown from the sensitivity analysis is the trend toward better outcomes regarding the 30-day/in-hospital mortality, survival and freedom from reintervention in studies reporting on PMSG. This may be explained by the fact that in recent years, increased technical skills and experience in advanced endovascular procedures have been gained in several centers to expand the applicability of the standard platforms.

The inherent limitations of a meta-analysis study, that is, heterogeneity among studies, also applied to our study. A selection bias may have also been introduced because of the retrospective nature of the eligible studies, while the reporting differences may have led to reporting bias and compromised the validity of the results. In addition, some studies did not differentiate outcomes between fenestrations, branches, urgency of repair or aneurysm extent, which limited further analysis. Moreover, follow-up periods were not homogeneous between the studies and were limited to 3 years, which precluded accurate medium- and long-term data analysis at specific time points. However, in order to overcome the significant differences between follow-up periods among eligible studies, we have reported IR per 100 p-ys instead of crude rates.

## 5. Conclusions

According to the present data, consisting mainly of retrospective studies, F/BEVAR can be performed in PDTAAAs with high rates of technical success and good mid-term results in terms of mortality and morbidity. The additional technical challenges posed by PDTAAAs need to be considered as part of meticulous planning in order to prevent complications and to decrease the considerable rate of reinterventions. Extensive experience in complex aortic endovascular repair and a strict follow-up regimen are valuable for achieving favorable and durable results in this subset of patients.

## Figures and Tables

**Table 1:** Baseline characteristics of the 10 eligible studies included in the meta-analysis.

Author	Study Design	Study Period	*n*	Stent Graft Design	F-UP (Months)	p-Years
**Abdelhalim, M.A., et al., 2023** [32]	retrospective, multicenter	2008–2021	246	209 CMD Cook, 37 t-Branch	24	492.00
**Patrick, R.J., et al., 2023** [33]	retrospective, single-center	2013–2022	18	all PMSG	12	18.00
**DiBartolomeo, A.D., et al., 2023** [34]	retrospective, single-center	2015–2021	32	all PMSG	11.90	31.73
**Yang, G., et al., 2023** [35]	retrospective, single-center	2017–2020	72	all PMSG	39.20	235.20
**Marques de Marino, P., et al., 2022** [36]	retrospective, single-center	2010–2021	75	42 fenestrated, 5 branched, 28 combined CMD	29	181.25
**Benfor, B., et al., 2022** [37]	retrospective, single-center	2017–2020	30	28 CMD, 2 off-the-shelf	15	37.50
**Wang, X., et al., 2022** [38]	retrospective, single-center	2014–2020	39	all PMS	29.4	95.55
**Gallitto, E., et al., 2022** [39]	retrospective, multicenter	2008–2019	37	33 CMD, 4 off-the-shelf	32	98.67
**Verzini, F., et al., 2020** [40]	retrospective, multicenter	2012–2018	21	18 CMD, 2 T-branch,1PMSG	23	40.25
**Kitagawa, A., et al., 2013** [41]	retrospective, single-center	2006–2011	15	all CMD	20.4	25.50

CMD: custom-made device; PMSG: physician-modified stent graft.

**Table 2:** Demographics and baseline characteristics of the analyzed patients.

		95% CIs
Total patients (*n*)	585	
Age (years)	61.6	58.5–64.7
Gender (% male)	77.9	
Comorbidities (%)		
HTN	91.0	77.0–96.8
Nicotine consumption (active or in past)	52.3	41.2–63.2
CAD	60.2	56.4–63.9
CVD	8.2	4.6–14.4
COPD	20.0	13.5–28.6
CKD (III-V)	17.7	13.4–23.0
Hyperlipidemia	43.6	29.0–59.5
DM	12.7	7.1–21.8
Prior aortic procedure	79.5	72.9–84.8
TAAA max diameter (mm)	60.2	56.4–63.9
Residual Type A	28.7	21.2–37.6
Chronic type B	65.8	26.1–91.3
Procedure mode (%)		
Elective	78.4	55.8–91.3
Urgent	23.1	8.2–50.2
Emergent	2.9	1.6–5.3

HTN: hypertension; CAD: coronary artery disease; CVD: cerebrovascular disease; COPD: chronic obstructive disease; CKD: chronic kidney disease; DM: diabetes mellitus.

**Table 3:** Sensitivity analysis regarding the design of the used stent grafts (manufactured or physician-modified).

	Pooled Estimation	95% CI	I^2^ (%)
**Technical success**			
Overall	94.3%	91.4% to 96.2%	10.9
Excluding studies on CMD + off-the-shelf SG	94.1%	88.1% to 97.2%	23.5
Excluding studies on PMSG	94.4%	91.6% to 96.4%	18.9
**30-day/in-hospital mortality**			
Overall	2.7%	1.6% to 4.4%	0.00
Excluding studies on CMD + off-the-shelf SG	2.0%	0.6% to 6.0%	0.0
Excluding studies on PMSG	2.9%	1.6% to 5.0%	0.0
**12-month survival**			
Overall	90.0%	86.8% to 92.5%	5.2
Excluding studies on CMD + off-the-shelf SG	93.6%	84.3% to 97.5%	42.7
Excluding studies on PMSG	89.3%	85.9% to 92.0%	0.0
**12-month TV patency**			
Overall	97.6%	94.9% to 98.8%	0.0
Excluding studies on CMD + off-the-shelf SG	98.0%	93.8% to 99.4%	0.0
Excluding studies on PMSG	97.2%	92.9% to 98.9%	0.0
**12-month freedom from reintervention**			
Overall	83.9%	75.9% to 89.6%	71.4
Excluding studies on CMD + off-the-shelf SG	89.2%	73.7% to 96.0%	73.2
Excluding studies on PMSG	80.5%	69.3% to 88.3%	75.2

## Data Availability

Data is contained within the manuscript and its Supplementary Materials.

## Supplementary Materials

The following supporting information can be downloaded at https://www.mdpi.com/article/10.3390/jcm13020410/s1. Supplementary Table S1: Study quality assessment with JBI checklist; Supplementary Table S2: Details on TV origin and modality of incorporation. Figure S1: Forest plot for Technical success. Figure S2: Forest plot for 30-day in-hospital mortality. Figure S3: Forest plot for Permanent paraplegia. Figure S4: Forest plot for Cerebrovascular events. Figure S5: Forest plot for Respiratory failure. Figure S6: Forest plot for Mesenteric ischaemia. Figure S7: Forest plot for Renal function impairment requiring new onset dialysis. Figure S8: Forest plot for MI. Figure S9: Forest plot for 12-month survival. Figure S10: Forest plot for 24-month survival. Figure S11: Forest plot for 36-month survival. Figure S12: Forest plot for 12-month patency. Figure S13: Forest plot for 24-month patency. Figure S14: Forest plot for 36-month patency. Figure S15: Forest plot for 12-month Freedom from reintervention. Figure S16: Forest plot for 24-month Freedom from reintervention. Figure S17: Forest plot for 36-month Freedom from reintervention. Figure S18: Forest plot for Reinterventions IR per 100 p-ys. FigureS19: Forest plot for Type I-III IR per 100 p-ys. File S1: PRISMA checklist References [32,33,34,35,36,37,38,39,40,41,50] are cited in Supplementary Materials.
