# Supplementary material for: A Systematic Review and an Updated Meta-Analysis of Fenestrated/Branched Endovascular Aortic Repair of Chronic Post-Dissection Thoracoabdominal Aortic Aneurysms
Source: J Clin Med. 2024 Jan 11;13(2):410. doi: 10.3390/jcm13020410 (PMC10816959; doi:10.3390/jcm13020410)
Supplement: Supplementary file 1 [file jcm-13-00410-s001.zip › jcm-2795434-supplementary.pdf]

PRISMA 2020 Checklist

Section and Topic	Item #	Checklist item	Location where item is reported
**TITLE**			Page #2
Title	1	Identify the report as a systematic review.	
**ABSTRACT**			
Abstract	2	See the PRISMA 2020 for Abstracts checklist.	Page #1
**INTRODUCTION**			
Rationale	3	Describe the rationale for the review in the context of existing knowledge.	Page #1-2
Objectives	4	Provide an explicit statement of the objective(s) or question(s) the review addresses.	Pages #2
**METHODS**			
Eligibility criteria	5	Specify the inclusion and exclusion criteria for the review and how studies were grouped for the syntheses.	Pages #2-3
Information sources	6	Specify all databases, registers, websites, organisations, reference lists and other sources searched or consulted to identify studies. Specify the date when each source was last searched or consulted.	Page #2-3
Search strategy	7	Present the full search strategies for all databases, registers and websites, including any filters and limits used.	Page #2-3
Selection process	8	Specify the methods used to decide whether a study met the inclusion criteria of the review, including how many reviewers screened each record and each report retrieved, whether they worked independently, and if applicable, details of automation tools used in the process.	Pages #2-3
Data collection process	9	Specify the methods used to collect data from reports, including how many reviewers collected data from each report, whether they worked independently, any processes for obtaining or confirming data from study investigators, and if applicable, details of automation tools used in the process.	Page #3
Data items	10a	List and define all outcomes for which data were sought. Specify whether all results that were compatible with each outcome domain in each study were sought (e.g. for all measures, time points, analyses), and if not, the methods used to decide which results to collect.	Pages #3
	10b	List and define all other variables for which data were sought (e.g. participant and intervention characteristics, funding sources). Describe any assumptions made about any missing or unclear information.	Pages #3
Study risk of bias assessment	11	Specify the methods used to assess risk of bias in the included studies, including details of the tool(s) used, how many reviewers assessed each study and whether they worked independently, and if applicable, details of automation tools used in the process.	Page #3
Effect measures	12	Specify for each outcome the effect measure(s) (e.g. risk ratio, mean difference) used in the synthesis or presentation of results.	Page #3
Synthesis methods	13a	Describe the processes used to decide which studies were eligible for each synthesis (e.g. tabulating the study intervention characteristics and comparing against the planned groups for each synthesis (item #5)).	Pages #2-3
	13b	Describe any methods required to prepare the data for presentation or synthesis, such as handling of missing summary statistics, or data conversions.	Page #3
	13c	Describe any methods used to tabulate or visually display results of individual studies and syntheses.	Page #3
	13d	Describe any methods used to synthesize results and provide a rationale for the choice(s). If meta-analysis was performed, describe the model(s), method(s) to identify the presence and extent of statistical heterogeneity, and software package(s) used.	Page #3
	13e	Describe any methods used to explore possible causes of heterogeneity among study results (e.g. subgroup analysis, meta-regression).	Page #3
	13f	Describe any sensitivity analyses conducted to assess robustness of the synthesized results.	Page #3
Reporting bias assessment	14	Describe any methods used to assess risk of bias due to missing results in a synthesis (arising from reporting biases).	
Certainty assessment	15	Describe any methods used to assess certainty (or confidence) in the body of evidence for an outcome.	Page #3

PRISMA 2020 Checklist

Section and Topic	Item #	Checklist item	Location where item is reported
**RESULTS**			
Study selection	16a	Describe the results of the search and selection process, from the number of records identified in the search to the number of studies included in the review, ideally using a flow diagram.	Pages #4-7
	16b	Cite studies that might appear to meet the inclusion criteria, but which were excluded, and explain why they were excluded.	Figure 1
Study characteristics	17	Cite each included study and present its characteristics.	Table 1
Risk of bias in studies	18	Present assessments of risk of bias for each included study.	Suppl Table 1
Results of individual studies	19	For all outcomes, present, for each study: (a) summary statistics for each group (where appropriate) and (b) an effect estimate and its precision (e.g. confidence/credible interval), ideally using structured tables or plots.	Table 1, Suppl Table 2
Results of syntheses	20a	For each synthesis, briefly summarise the characteristics and risk of bias among contributing studies.	Pages 4-8<br>Suppl Figures #1-19
	20b	Present results of all statistical syntheses conducted. If meta-analysis was done, present for each the summary estimate and its precision (e.g. confidence/credible interval) and measures of statistical heterogeneity. If comparing groups, describe the direction of the effect.	Pages 4-8<br>Suppl Figures #1-19
	20c	Present results of all investigations of possible causes of heterogeneity among study results.	Pages 4-8<br>Suppl Figures #1-19
	20d	Present results of all sensitivity analyses conducted to assess the robustness of the synthesized results.	Table 3
Reporting biases	21	Present assessments of risk of bias due to missing results (arising from reporting biases) for each synthesis assessed.	Not applicable
Certainty of evidence	22	Present assessments of certainty (or confidence) in the body of evidence for each outcome assessed.	Pages 4-8<br>Suppl Figures #1-19
**DISCUSSION**			
Discussion	23a	Provide a general interpretation of the results in the context of other evidence.	Page #8
	23b	Discuss any limitations of the evidence included in the review.	Pages #9
	23c	Discuss any limitations of the review processes used.	Page #9
	23d	Discuss implications of the results for practice, policy, and future research.	Page 9
**OTHER INFORMATION**			
Registration and	24a	Provide registration information for the review, including register name and registration number, or state that the review was not registered.	Page #2

Section and Topic	Item #	Checklist item	Location where item is reported
protocol	24b	Indicate where the review protocol can be accessed, or state that a protocol was not prepared.	PROSPERO public database
	24c	Describe and explain any amendments to information provided at registration or in the protocol.	Not applicable
Support	25	Describe sources of financial or non-financial support for the review, and the role of the funders or sponsors in the review.	Page #9
Competing interests	26	Declare any competing interests of review authors.	Page #9
Availability of data, code and other materials	27	Report which of the following are publicly available and where they can be found: template data collection forms; data extracted from included studies; data used for all analyses; analytic code; any other materials used in the review.	Page #9

	1. Were there clear criteria for inclusion in the case series?	2. Was the condition measured in a standard, reliable way for all participants included in the case series?	3. Were valid methods used for identification of the condition for all participants included in the case series?	4. Did the case series have consecutive inclusion of participants?	5. Did the case series have complete inclusion of participants?	6. Was there clear reporting of the demographics of the participants in the study?	7. Was there clear reporting of clinical information of the participants?	8. Were the outcomes or follow-up results of cases clearly reported?	9. Was there clear reporting of the presenting site(s)/clinic(s) demographic information?	10. Was statistical analysis appropriate?
Abdelhalim MA, et al.2023	Yes	Yes	Yes	Yes	Yes	Yes	Yes	Yes	Not appl.	Yes
Patrick RJ, et al. 2023	Yes	Yes	Yes	Yes	Yes	Yes	Yes	Yes	Not appl.	Yes
DiBartolomeo AD, et al. 2023	Yes	Yes	Yes	Yes	Yes	Yes	Yes	Yes	Not appl.	Yes
Yang G, et al. 2023	Yes	Yes	Yes	Yes	Yes	Yes	Yes	Yes	Not appl.	Yes
de Marino PM, et al. 2022	Yes	Yes	Yes	Yes	Yes	Yes	Yes	Yes	Not appl.	Yes
Benfor B, et al. 2022	Yes	Yes	Yes	Yes	Yes	Yes	Yes	Yes	Not appl.	Yes
Wang X, et al. 2022	Yes	Yes	Yes	Yes	Yes	Yes	Yes	Yes	Not appl.	Yes
Gallitto E, et al. 2022	Yes	Yes	Yes	Yes	Yes	Yes	Yes	Yes	Not appl.	Yes
Verzini F, et al. 2020	Yes	Yes	Yes	Yes	Yes	Yes	Yes	Yes	Not appl.	Yes
Kitagawa A, et al. 2013	Yes	Yes	Yes	Yes	Yes	Yes	Yes	Yes	Not appl.	Yes

Supplementary Table S1: Study quality assessment with JBI checklist

Supplementary Table S2: Details on TV origin and modality of incorporation

Author	Target vessels					Origin			Modality of TV incorporation				Perforation of the flap needed (n of vessels)
	Total	RAs	SMA	CT	other	TL	FL	Both	fens	dir. branches	inner branches	scallops	
**Abdelhalim MA, et al.2023[1]**	917	443	241	225	8	NR	NR	NR	581	336	0	0	NR
**Patrick RJ, et al. 2023[2]**	71	36	18	17	0	54	15	2	0	71	0	0	2
**DiBartolomeo AD, et al. 2023[3]**	132	64	30	32	6	NR	NR	NR	105	11	5	2	0
**Yang G, et al. 2023[4]**	234	NR	NR	NR	NR	NR	NR	NR	NR	NR	NR	NR	12
**Marques de Marino P, et al. 2022[5]**	NR	NR	NR	NR	NR	NR	NR	NR	NR	NR	NR	NR	NR
**Benfor B, et al. 2022[6]**	116	57	30	29	0	NR	NR	NR	92	25	2	0	NR
**Wang X, et al. 2022[7]**	126	76	35	15	0	94	14	18	126	0	0	0	NR
**Gallitto E, et al. 2022[8]**	135	66	46	33	0	120	7	8	96	39	0	0	NR
**Verzini F, et al. 2020[9]**	84	42	21	20	1	77	7	2	72	12	0	0	5
**Kitagawa A, et al. 2013[10]**	60	30	15	15	0	NR	NR	NR	NR	NR	NR	NR	NR

RAs: renal arteries; SMA: superior mesenteric artery; CT: coeliac trunk; TL: true lumen; FL: false lumen; fens: fenestrations; dir. branches: directional branches

1. Abdelhalim MA, Tenorio ER, Oderich GS, Haulon S, Warren G, Adam D, et al. Multicenter trans-Atlantic experience with fenestrated-branched endovascular aortic repair of chronic post-dissection thoracoabdominal aortic aneurysms. J Vasc Surg. 2023;78(4):854-62 e1.
2. Patrick RJ, Patrick R, Lucas SJ, VandenHull A, Reed V, Sengos J, et al. Treatment of Thoracoabdominal Aortic Aneurysmal Degeneration Following Aortic Dissections at a Single Surgical Center Using a Physician-Assembled Branched Endovascular Stent Graft. Annals of Vascular Surgery. 2023;93:174-84.
3. DiBartolomeo AD, Pyun AJ, Ding L, O'Donnell K, Paige JK, Magee GA, et al. Comparative outcomes of physician-modified fenestrated-branched endovascular repair of post-dissection and degenerative complex abdominal or thoracoabdominal aortic aneurysms. J Vasc Surg. 2023;78(3):565-74 e2.
4. Yang G, Zhang M, Zhang Y, Wu G, Li X, Zhou M. Midterm outcomes of physician-modified endovascular grafts for repair of postdissection and degenerative thoracoabdominal aortic aneurysms. JTCVS Tech. 2023;18:1-10.
5. Marques de Marino P, Ibraheem A, Tesinsky P, Abu Jiries M, Katsargyris A, Verhoeven EL. Fenestrated and branched stent grafts for the treatment of post-dissection thoracoabdominal aortic aneurysms. Semin Vasc Surg. 2022;35(3):312-9.
6. Benfor B, Hogl J, Gouveia EMR, Stana J, Prendes CF, Pichlmaier M, et al. Postoperative Outcomes and Reinterventions Following Fenestrated/Branched Endovascular Aortic Repair in Post-Dissection and Complex Degenerative Abdominal and Thoraco-Abdominal Aortic Aneurysms. J Clin Med. 2022;11(16).
7. Wang X, Zhu Q, He Y, Shang T, Xiang Y, Zeng Q, et al. Mid-term Outcomes of Physician-Modified Fenestrated or Branched Endovascular Repair for Post-dissection Thoracoabdominal Aortic Aneurysms. Cardiovasc Intervent Radiol. 2022;45(11):1672-81.
8. Gallitto E, Faggioli G, Melissano G, Fargion A, Isernia G, Bertoglio L, et al. Fenestrated and Branched Endografts for Post-Dissection Thoraco-Abdominal Aneurysms: Results of a National Multicentre Study and Literature Review. Eur J Vasc Endovasc Surg. 2022;64(6):630-8.
9. Verzini F, Ferrer C, Parlani G, Coscarella C, Giudice R, Frola E, et al. Mid-Term Outcomes of Complex Endografting for Chronic Post-Dissection Thoracoabdominal Aortic Aneurysms. Cardiovasc Intervent Radiol. 2020;43(10):1440-8.
10. Kitagawa A, Greenberg RK, Eagleton MJ, Mastracci TM, Roselli EE. Fenestrated and branched endovascular aortic repair for chronic type B aortic dissection with thoracoabdominal aneurysms. J Vasc Surg. 2013;58(3):625-34.

Figure S1: Technical success

Study name	Event rate	Lower limit	Upper limit
Abdelhalim MA, et al.2023	0.955	0.921	0.975
Patrick RJ, et al. 2023	0.944	0.693	0.992
DiBartolomeo AD, et al. 2023	0.969	0.809	0.996
Yang G, et al. 2023	0.986	0.908	0.998
Marques de Marino P, et al. 2022	0.987	0.911	0.998
Benfor B, et al. 2022	0.933	0.769	0.983
Wang X, et al. 2022	0.897	0.757	0.961
Gallitto E, et al. 2022	0.919	0.777	0.974
Verzini F, et al. 2020	0.857	0.639	0.953
Kitagawa A, et al. 2013	0.969	0.650	0.998
Pooled	0.943	0.914	0.962
Prediction Interval	0.943	0.887	0.972

Event rate and 95% CI

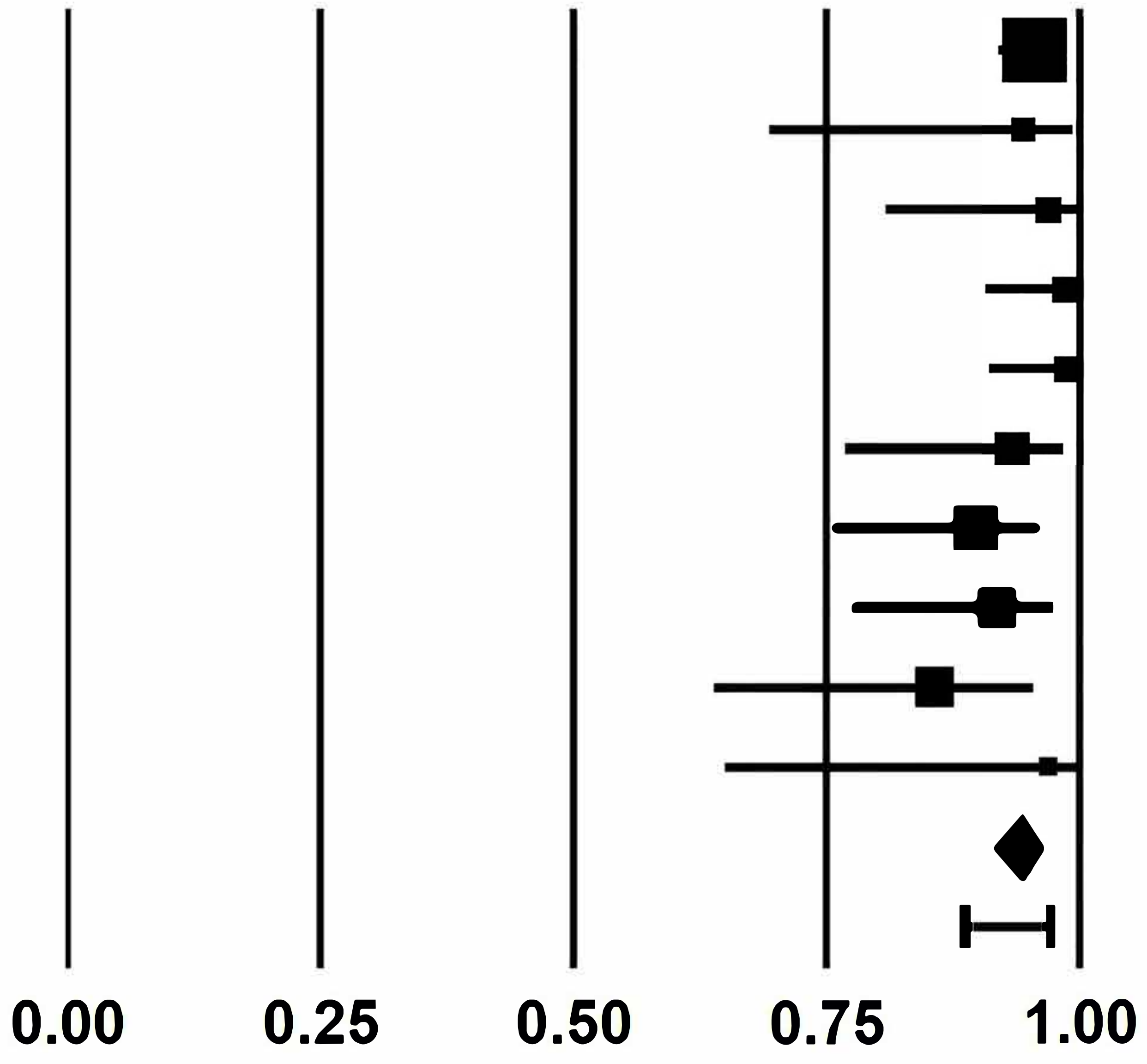

The I-squared statistic is 10.9%, p 0.341

Figure S2: 30-day/in-hospital mortality

Study name	Event rate	Lower limit	Upper limit
Abdelhalim MA, et al.2023	0.033	0.016	0.064
Patrick RJ, et al. 2023	0.026	0.002	0.310
DiBartolomeo AD, et al. 2023	0.031	0.004	0.191
Yang G, et al. 2023	0.014	0.002	0.092
Marques de Marino P, et al. 2022	0.027	0.007	0.100
Benfor B, et al. 2022	0.016	0.001	0.211
Wang X, et al. 2022	0.013	0.001	0.171
Gallitto E, et al. 2022	0.013	0.001	0.178
Verzini F, et al. 2020	0.023	0.001	0.277
Kitagawa A, et al. 2013	0.031	0.002	0.350
Pooled	0.027	0.016	0.044

Statistics for each study

Event rate and 95% CI

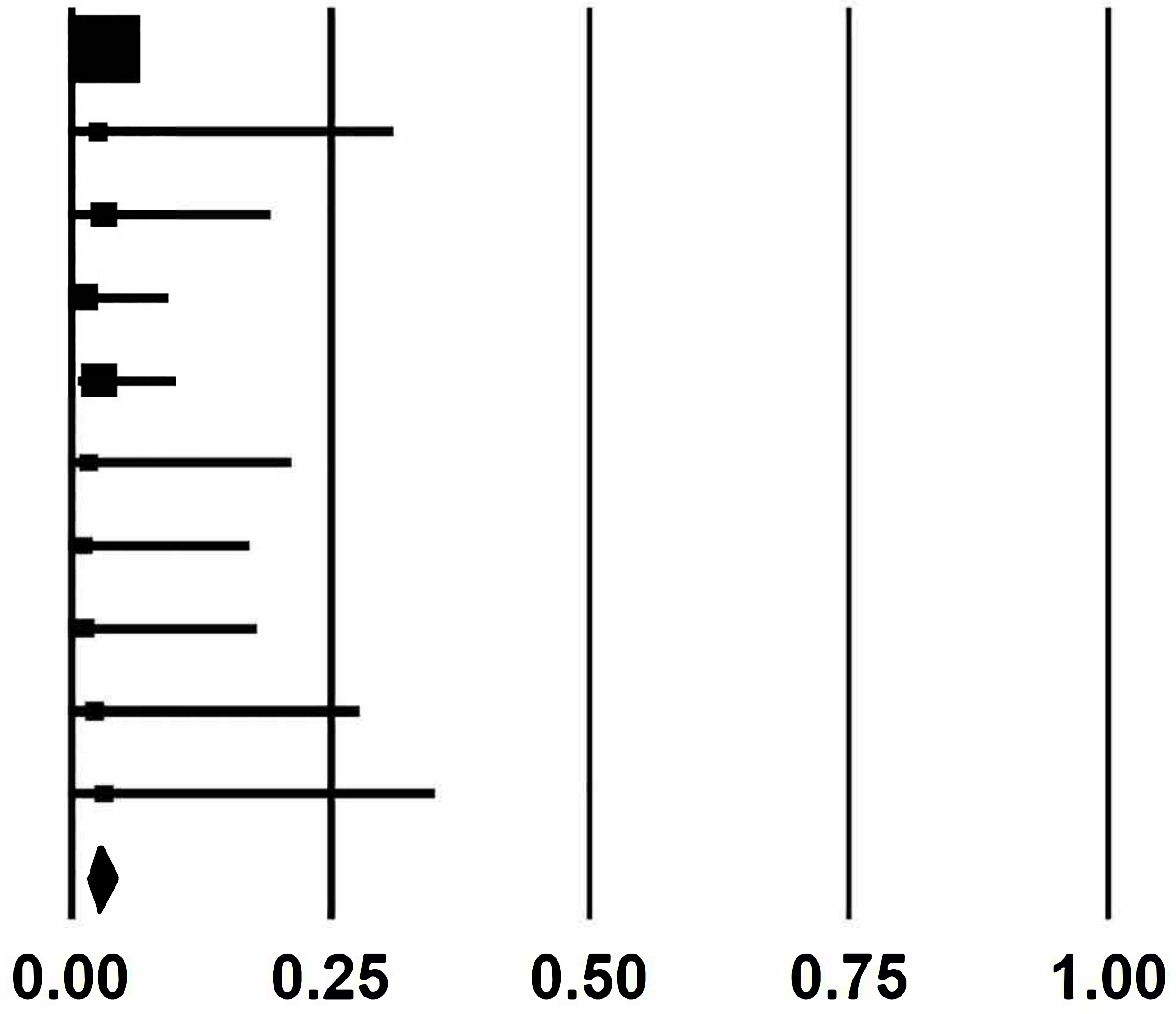

The I-squared statistic is 0% p0.997

Figure S3: Permanent paraplegia

Study name	Event rate	Lower limit	Upper limit
Abdelhalim MA, et al.2023	0.024	0.011	0.053
Patrick RJ, et al. 2023	0.026	0.002	0.310
DiBartolomeo AD, et al. 2023	0.063	0.016	0.218
Yang G, et al. 2023	0.007	0.000	0.100
Marques de Marino P, et al. 2022	0.013	0.002	0.089
Benfor B, et al. 2022	0.033	0.005	0.202
Wang X, et al. 2022	0.013	0.001	0.171
Gallitto E, et al. 2022	0.013	0.001	0.178
Verzini F, et al. 2020	0.023	0.001	0.277
Kitagawa A, et al. 2013	0.031	0.002	0.350
Pooled	0.025	0.015	0.043
Prediction Interval			

Event rate and 95% CI

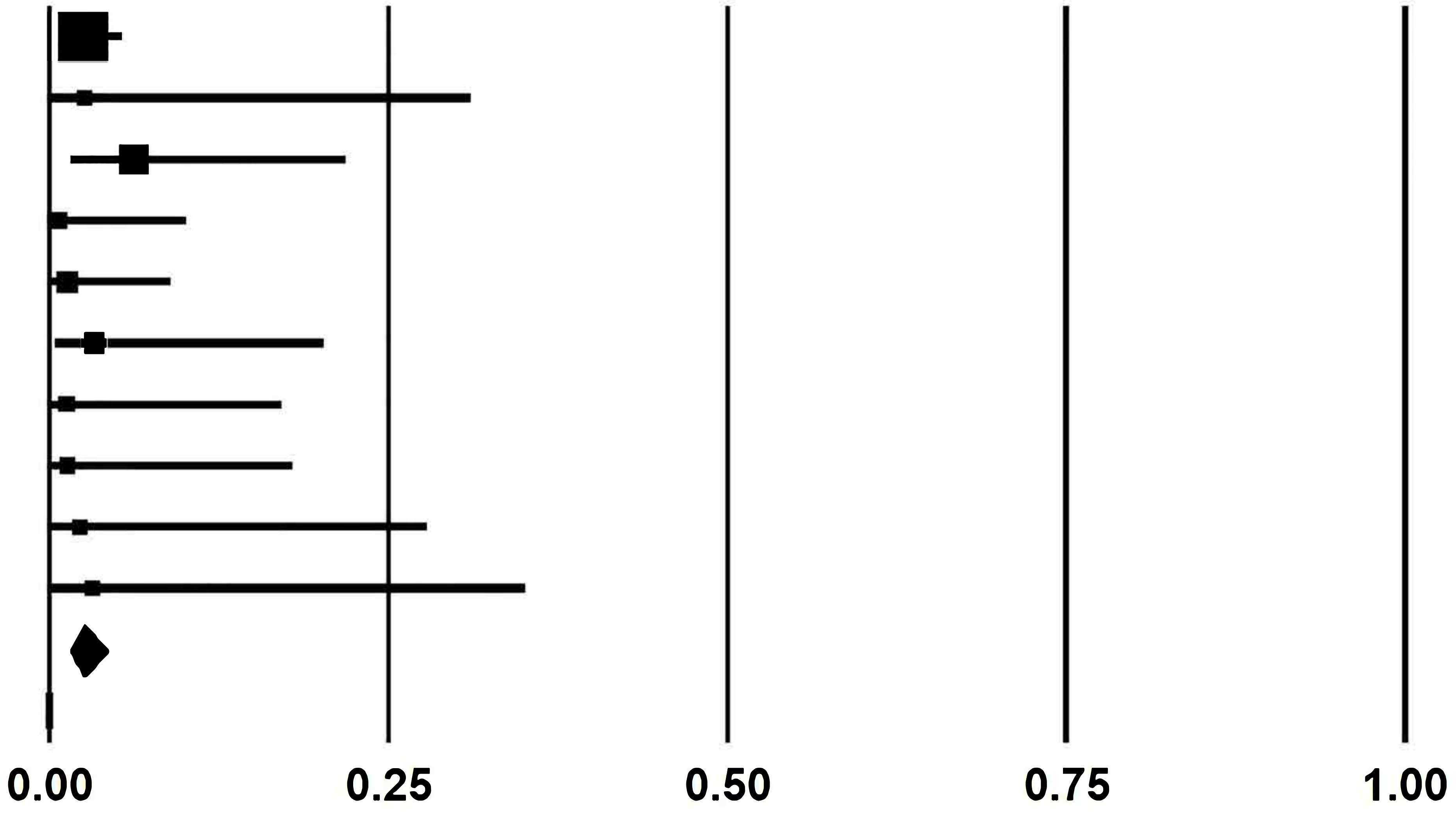

The I-squared statistic is 0%, p 0.938

Figure S4: Cerebrovascular events (stroke/TIA)

Study name	Event rate	Lower limit	Upper limit
Abdelhalim MA, et al.2023	0.012	0.004	0.037
Patrick RJ, et al. 2023	0.026	0.002	0.310
DiBartolomeo AD, et al. 2023	0.031	0.004	0.191
Yang G, et al. 2023	0.014	0.002	0.092
Marques de Marino P, et al. 2022	0.007	0.000	0.097
Benfor B, et al. 2022	0.016	0.001	0.211
Wang X, et al. 2022	0.013	0.001	0.171
Gallitto E, et al. 2022	0.013	0.001	0.178
Verzini F, et al. 2020	0.023	0.001	0.277
Kitagawa A, et al. 2013	0.031	0.002	0.350
Pooled	0.016	0.008	0.030
Prediction Interval			

Event rate and 95% CI

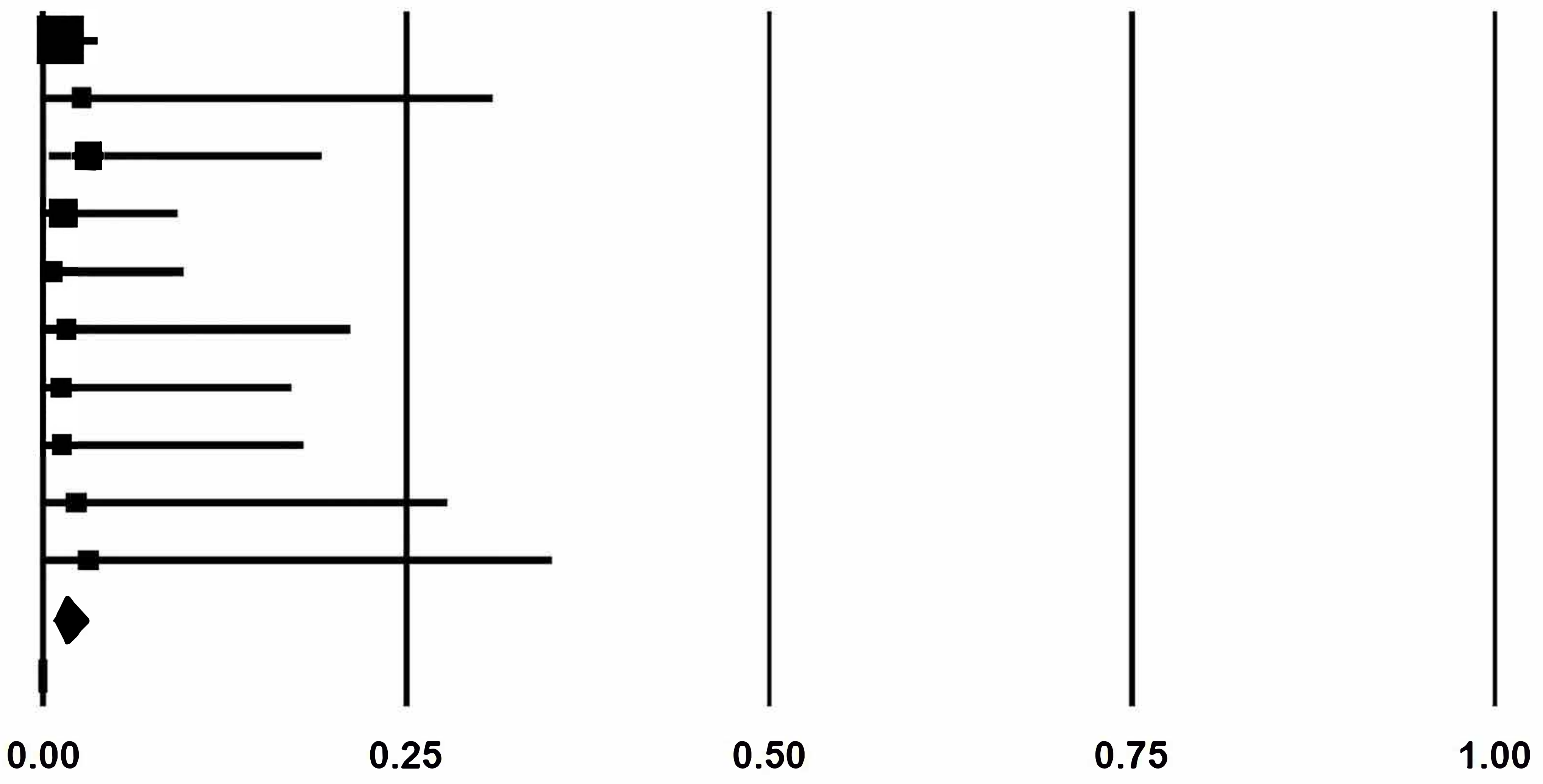

The I-squared statistic is 0% p 0.997

Figure S5: Respiratory failure

Study name	Event rate	Lower limit	Upper limit
Abdelhalim MA, et al.2023	0.061	0.037	0.099
Patrick RJ, et al. 2023	0.111	0.028	0.352
DiBartolomeo AD, et al. 2023	0.015	0.001	0.201
Yang G, et al. 2023	0.028	0.007	0.104
Marques de Marino P, et al. 2022	0.007	0.000	0.097
Benfor B, et al. 2022	0.033	0.005	0.202
Wang X, et al. 2022	0.013	0.001	0.171
Gallitto E, et al. 2022	0.108	0.041	0.255
Verzini F, et al. 2020	0.023	0.001	0.277
Kitagawa A, et al. 2013	0.031	0.002	0.350
Pooled	0.055	0.037	0.082
Prediction Interval	0.055	0.031	0.095

Event rate and 95% CI

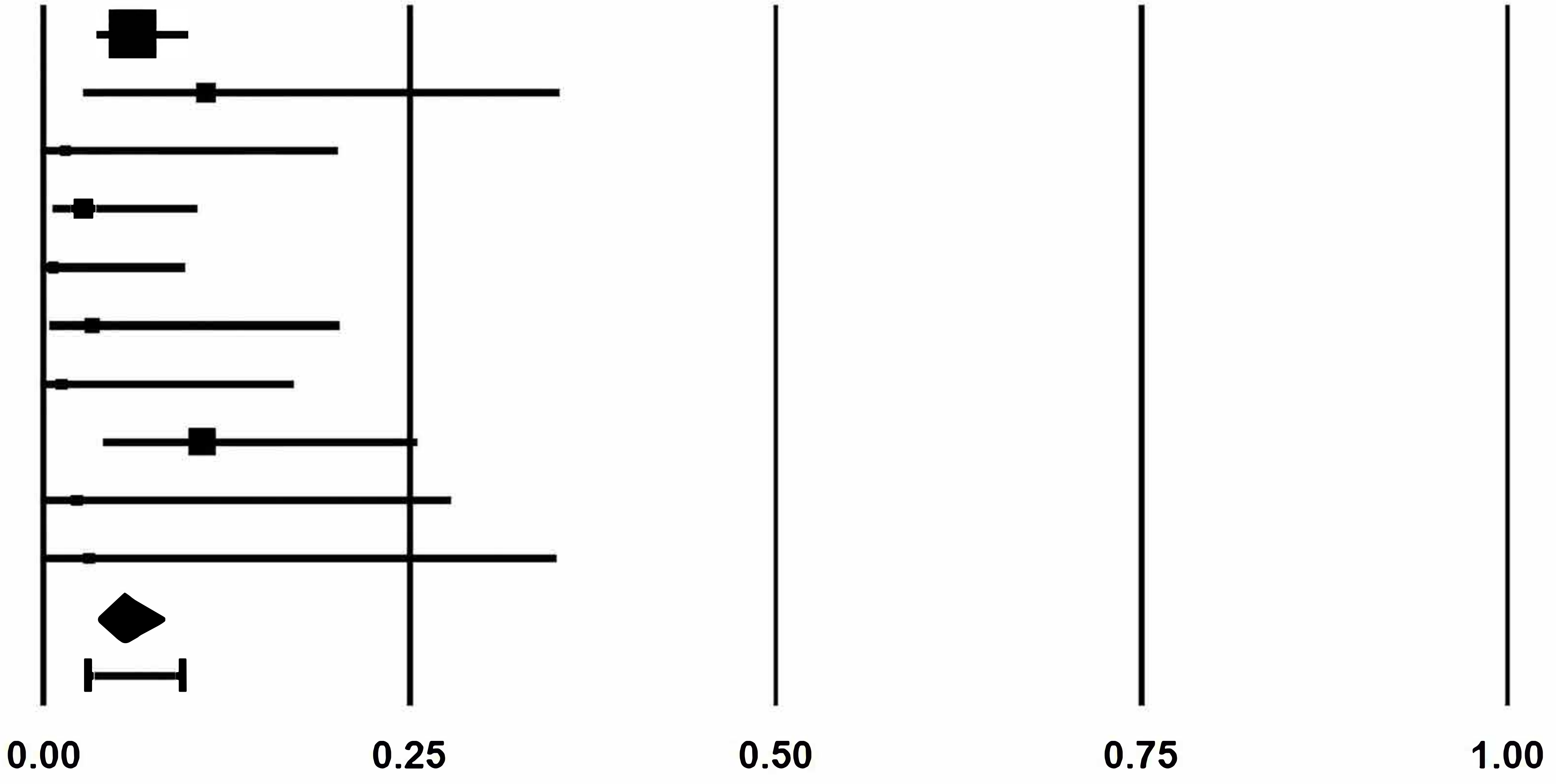

The I-squared statistic is 3.4% p 0.408

Figure S6: Mesenteric ischaemia

Study name	Event rate	Lower limit	Upper limit
Abdelhalim MA, et al.2023	0.004	0.001	0.028
Patrick RJ, et al. 2023	0.026	0.002	0.310
DiBartolomeo AD, et al. 2023	0.015	0.001	0.201
Yang G, et al. 2023	0.014	0.002	0.092
Marques de Marino P, et al. 2022	0.013	0.002	0.089
Benfor B, et al. 2022	0.016	0.001	0.211
Wang X, et al. 2022	0.013	0.001	0.171
Gallitto E, et al. 2022	0.013	0.001	0.178
Verzini F, et al. 2020	0.023	0.001	0.277
Kitagawa A, et al. 2013	0.031	0.002	0.350
Pooled	0.013	0.006	0.028
Prediction Interval			

Event rate and 95% CI

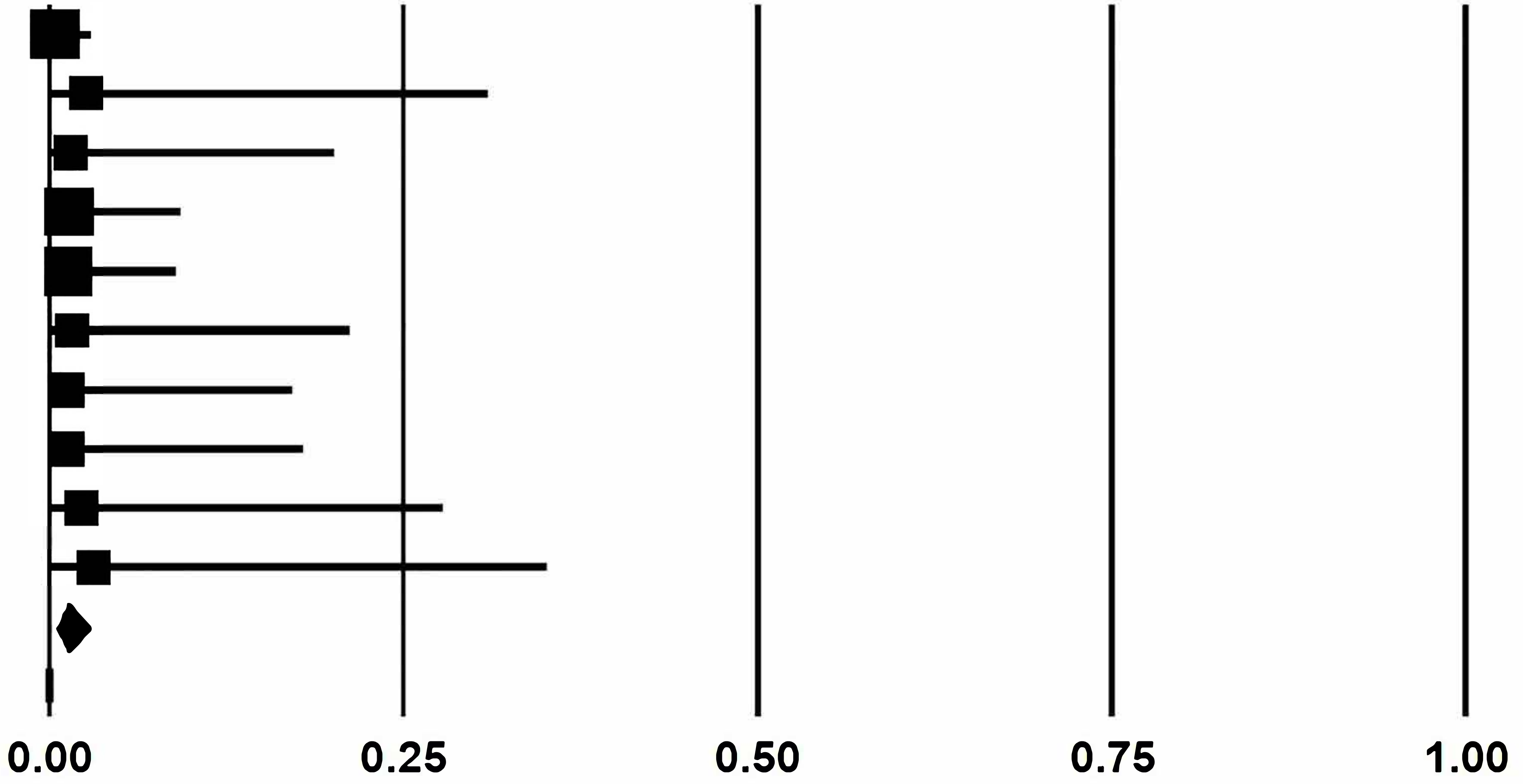

The I-squared statistic is 0%, p 0.988

Figure S7: Renal function impairment requiring new onset dialysis

Study name	Event rate	Lower limit	Upper limit
Abdelhalim MA, et al.2023	0.008	0.002	0.032
Patrick RJ, et al. 2023	0.026	0.002	0.310
DiBartolomeo AD, et al. 2023	0.015	0.001	0.201
Yang G, et al. 2023	0.014	0.002	0.092
Marques de Marino P, et al. 2022	0.007	0.000	0.097
Benfor B, et al. 2022	0.067	0.017	0.231
Wang X, et al. 2022	0.026	0.004	0.161
Gallitto E, et al. 2022	0.013	0.001	0.178
Verzini F, et al. 2020	0.023	0.001	0.277
Kitagawa A, et al. 2013	0.031	0.002	0.350
Pooled	0.020	0.010	0.038
Prediction Interval			

Event rate and 95% CI

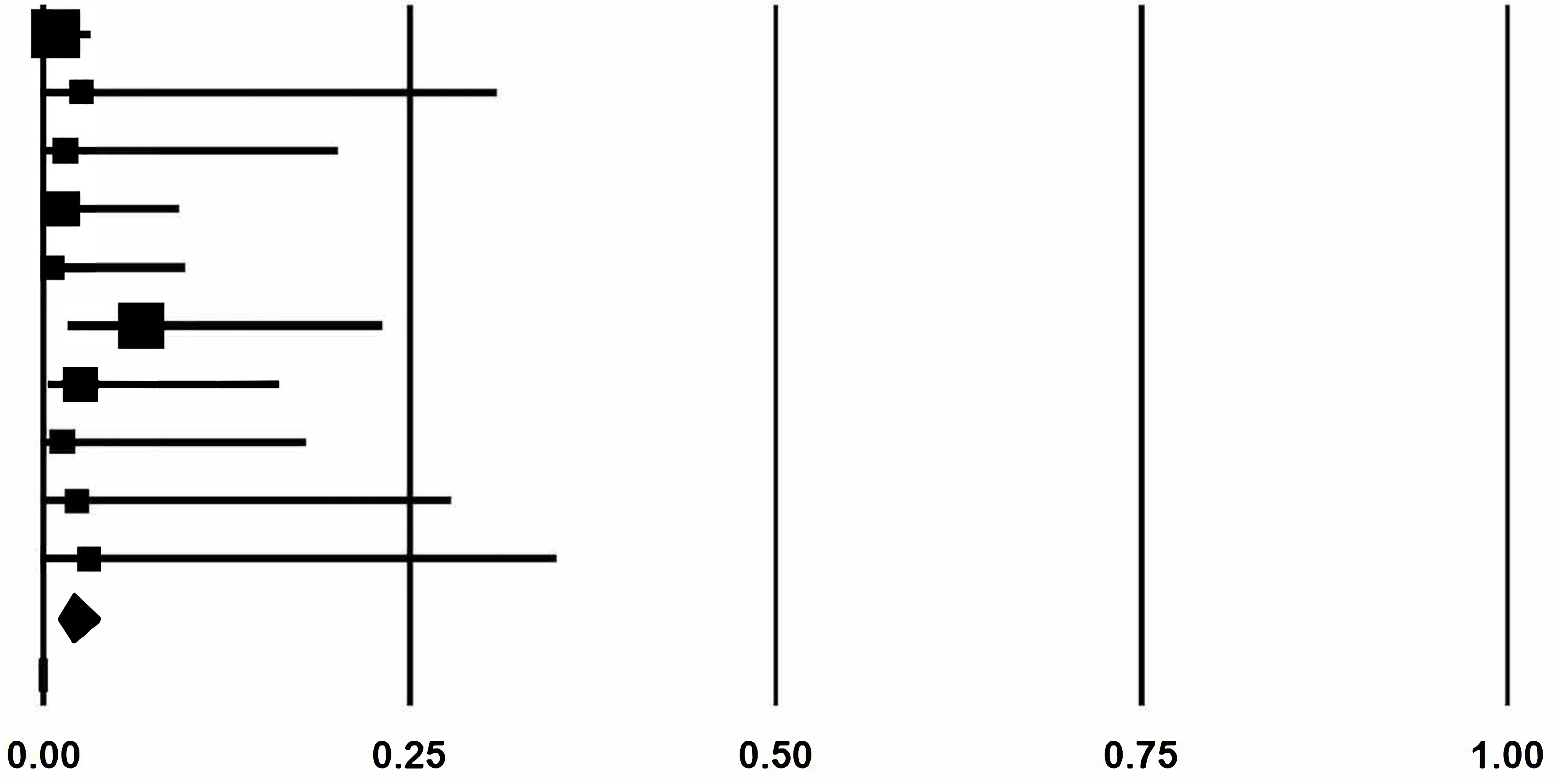

The I-squared statistic is 0% p 0.771

Figure S8: Myocardial infarction

Study name	Event rate	Lower limit	Upper limit
Abdelhalim MA, et al.2023	0.008	0.002	0.032
Patrick RJ, et al. 2023	0.026	0.002	0.310
DiBartolomeo AD, et al. 2023	0.015	0.001	0.201
Yang G, et al. 2023	0.014	0.002	0.092
Marques de Marino P, et al. 2022	0.007	0.000	0.097
Benfor B, et al. 2022	0.016	0.001	0.211
Wang X, et al. 2022	0.013	0.001	0.171
Gallitto E, et al. 2022	0.013	0.001	0.178
Verzini F, et al. 2020	0.048	0.007	0.271
Kitagawa A, et al. 2013	0.031	0.002	0.350
Pooled	0.015	0.007	0.030
Prediction Interval			

Event rate and 95% CI

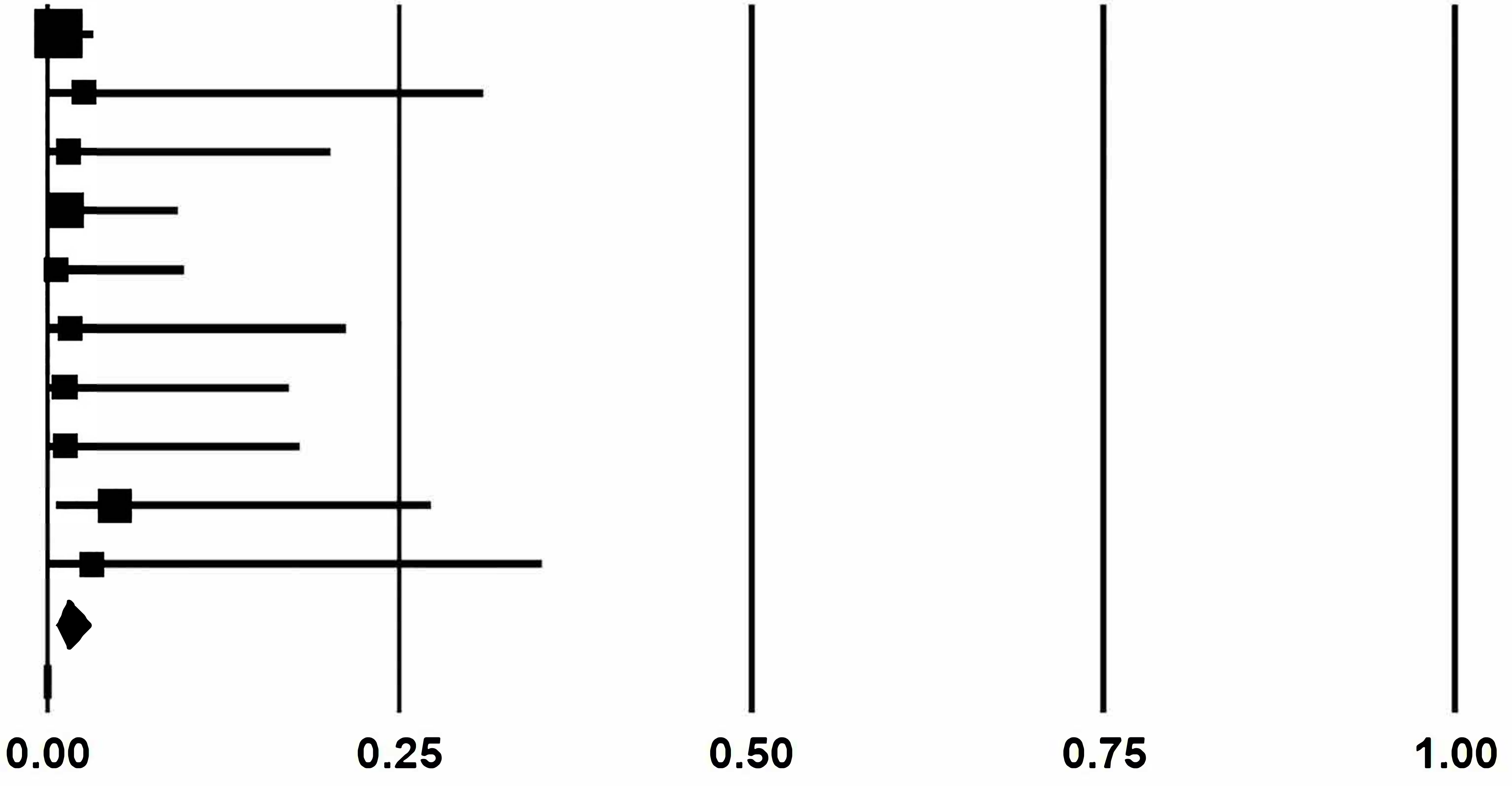

The I-squared statistic is 0%, p 0.967

Figure S9: 12-month Survival

Study name	Statistics for each study<br>Event rate	 Lower limit	 Upper limit
Abdelhalim MA, et al.2023	0.897	0.852	0.929
Patrick RJ, et al. 2023	0.889	0.648	0.972
DiBartolomeo AD, et al. 2023	0.875	0.711	0.952
Yang G, et al. 2023	0.972	0.895	0.993
Marques de Marino P, et al. 2022	0.870	0.773	0.929
Benfor B, et al. 2022	0.984	0.789	0.999
Wang X, et al. 2022	0.988	0.829	0.999
Gallitto E, et al. 2022	0.890	0.743	0.958
Verzini F, et al. 2020	0.938	0.720	0.989
Kitagawa A, et al. 2013	0.850	0.579	0.959
Pooled	0.900	0.868	0.925
Prediction Interval	0.900	0.850	0.935

Event rate and 95% CI

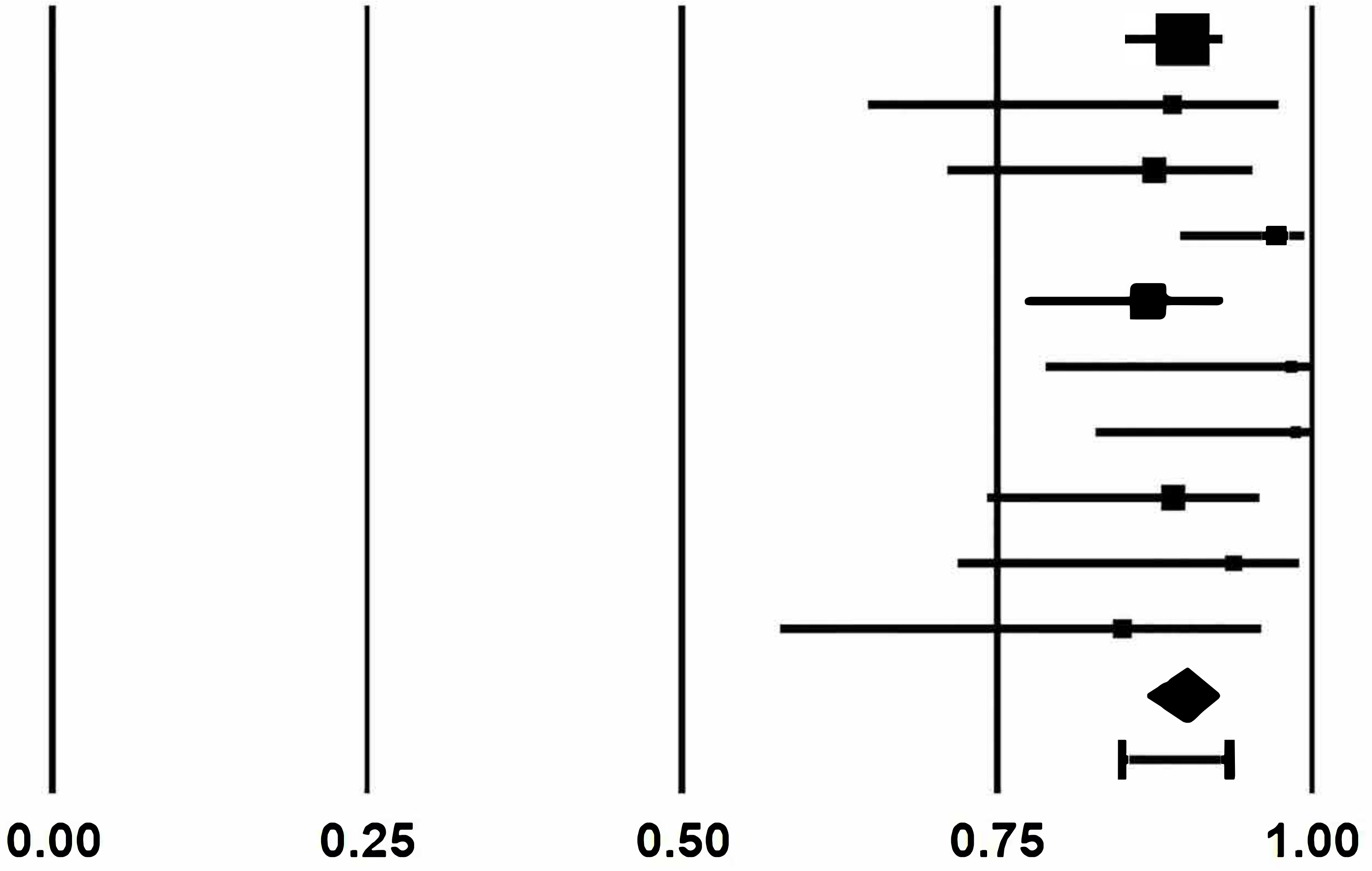

The I-squared statistic is 5.2% p0.393

Figure S10: 24-month Survival

Study name	Statistics for each study		
	Event rate	Lower limit	Upper limit
Abdelhalim MA, et al.2023	0.817	0.764	0.860
Yang G, et al. 2023	0.972	0.895	0.993
Marques de Marino P, et al. 2022	0.835	0.733	0.903
Wang X, et al. 2022	0.988	0.829	0.999
Gallitto E, et al. 2022	0.850	0.697	0.933
Verzini F, et al. 2020	0.938	0.720	0.989
Kitagawa A, et al. 2013	0.850	0.579	0.959
Pooled	0.878	0.809	0.925
Prediction Interval	0.878	0.640	0.967

Event rate and 95% CI

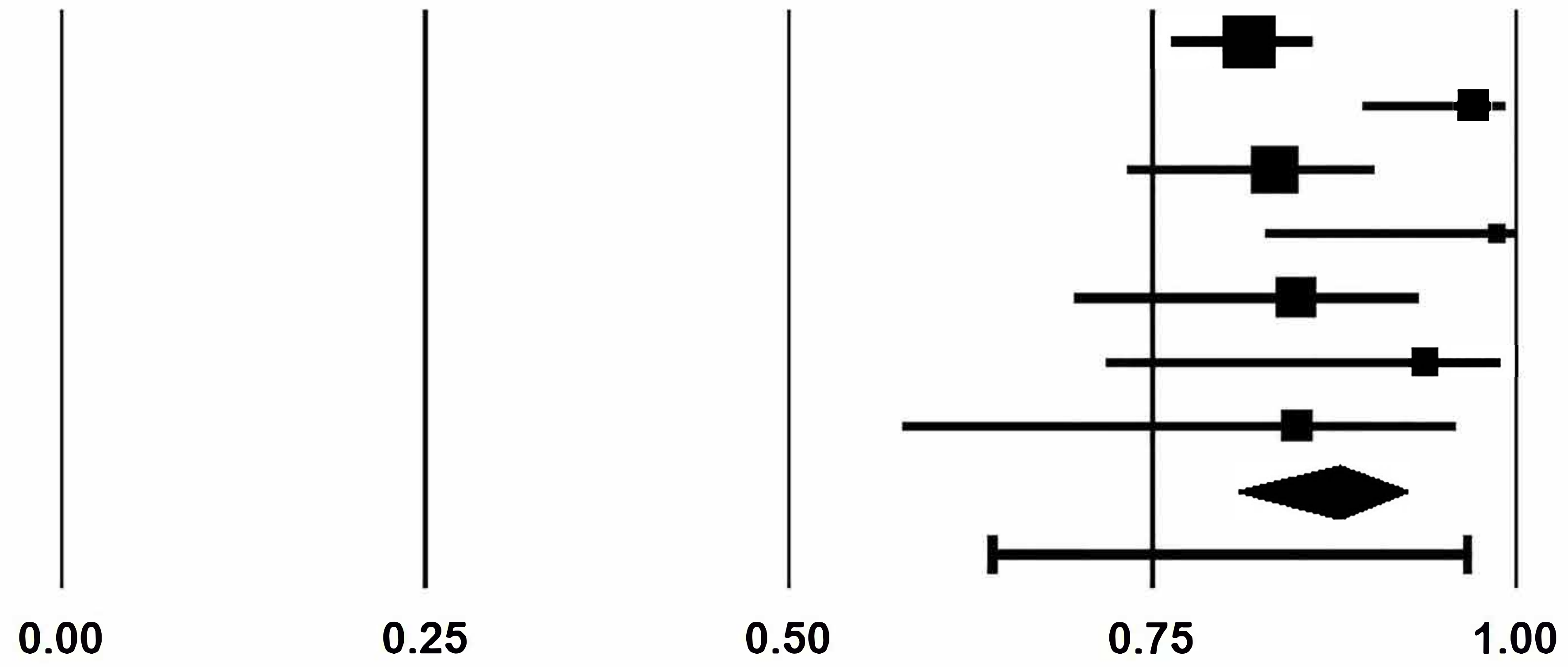

The I-squared statistic is 54% p0.043

Figure S11: 36-month Survival

Study name	Event rate	Lower limit	Upper limit
Abdelhalim MA, et al.2023	0.787	0.731	0.834
Yang G, et al. 2023	0.972	0.895	0.993
Marques de Marino P, et al. 2022	0.722	0.610	0.811
Wang X, et al. 2022	0.988	0.829	0.999
Gallitto E, et al. 2022	0.810	0.652	0.906
Verzini F, et al. 2020	0.938	0.720	0.989
Kitagawa A, et al. 2013	0.850	0.579	0.959
Pooled	0.855	0.765	0.915
Prediction Interval	0.855	0.522	0.970

(Column group heading: Statistics for each study)

Event rate and 95% CI

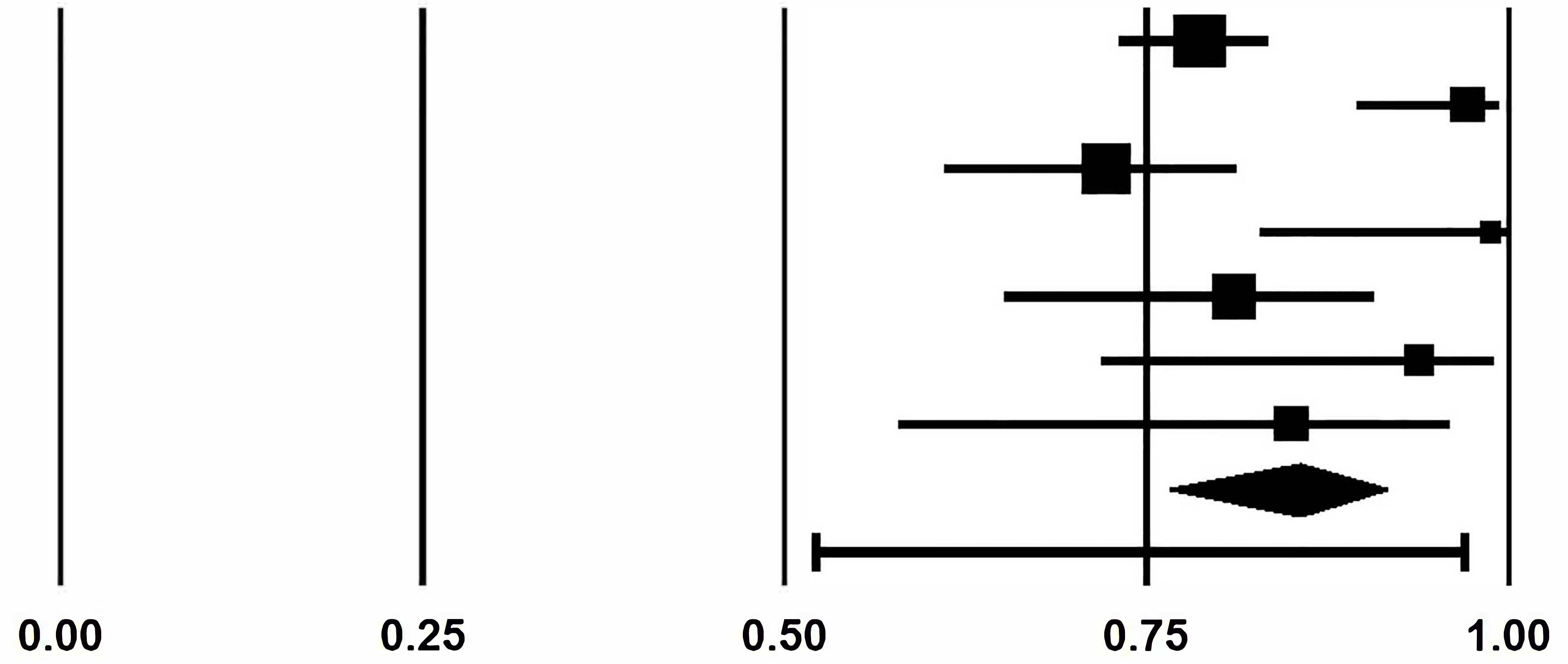

The I-squared statistic is 68.4% p0.004

Figure S12: 12-month TV Patency

Study name	Event rate	Lower limit	Upper limit
Patrick RJ, et al. 2023	0.986	0.580	1.000
DiBartolomeo AD, et al. 2023	0.969	0.809	0.996
Yang G, et al. 2023	0.991	0.905	0.999
Marques de Marino P, et al. 2022	0.979	0.906	0.996
Benfor B, et al. 2022	0.948	0.784	0.989
Wang X, et al. 2022	0.976	0.840	0.997
Gallitto E, et al. 2022	0.987	0.822	0.999
Verzini F, et al. 2020	0.977	0.723	0.999
Pooled	0.976	0.949	0.988
Prediction Interval			

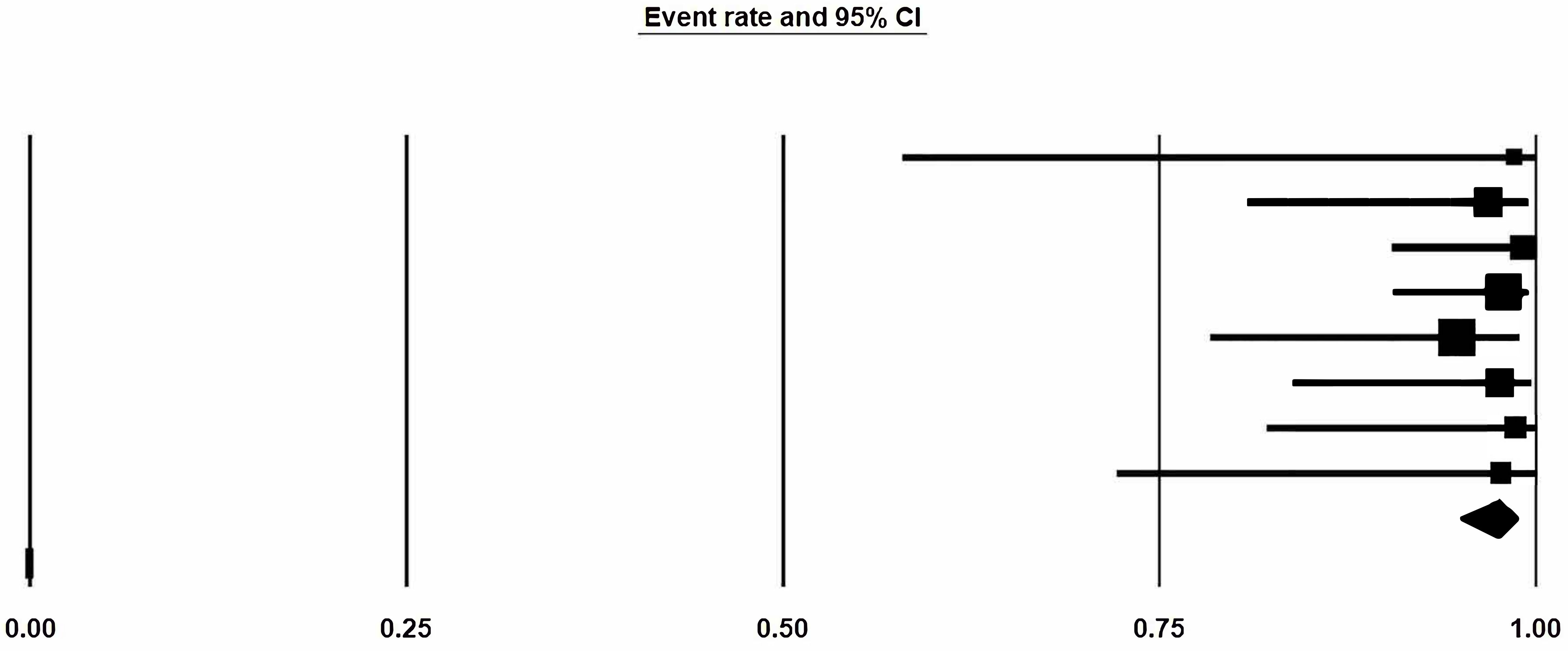

The I-squared statistic is 0% p0.963

Figure S13: 24-month TV Patency

Study name	Event rate	Lower limit	Upper limit
Yang G, et al. 2023	0.978	0.902	0.995
Marques de Marino P, et al. 2022	0.961	0.884	0.988
Wang X, et al. 2022	0.964	0.832	0.993
Gallitto E, et al. 2022	0.960	0.823	0.992
Verzini F, et al. 2020	0.977	0.723	0.999
Pooled	0.967	0.935	0.983
Prediction Interval			

Event rate and 95% CI

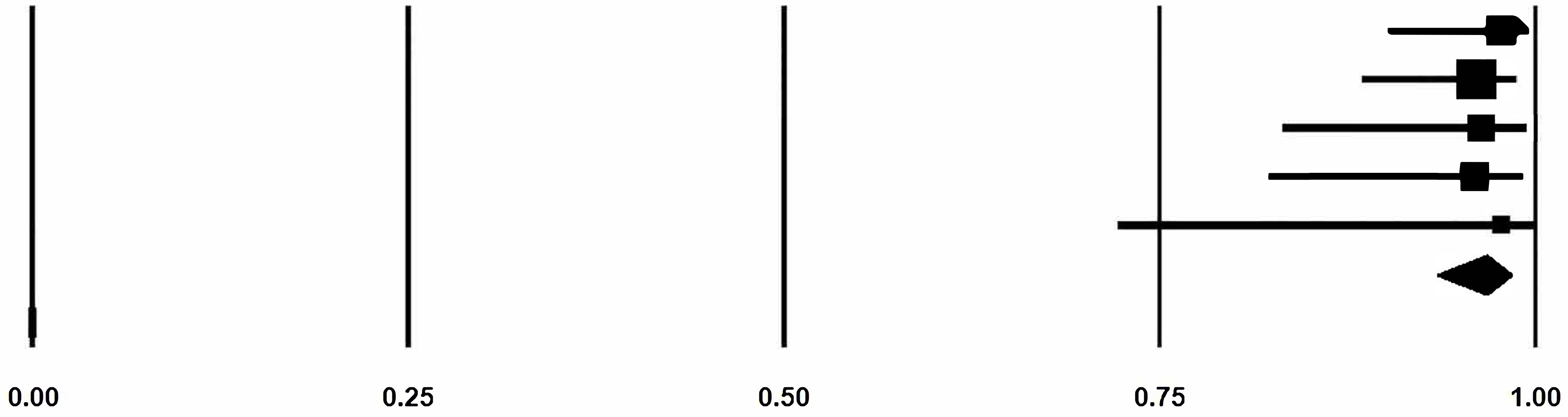

The I-squared statistic is 0% p0.974

Figure S14: 36-month TV Patency

Study name | **Event rate and 95% CI**

Study name	Event rate	Lower limit	Upper limit
Abdelhalim MA, et al.2023	0.930	0.891	0.956
Yang G, et al. 2023	0.973	0.897	0.993
Marques de Marino P, et al. 2022	0.952	0.873	0.983
Wang X, et al. 2022	0.964	0.832	0.993
Gallitto E, et al. 2022	0.960	0.823	0.992
Verzini F, et al. 2020	0.977	0.723	0.999
Pooled	0.943	0.918	0.961
Prediction Interval			

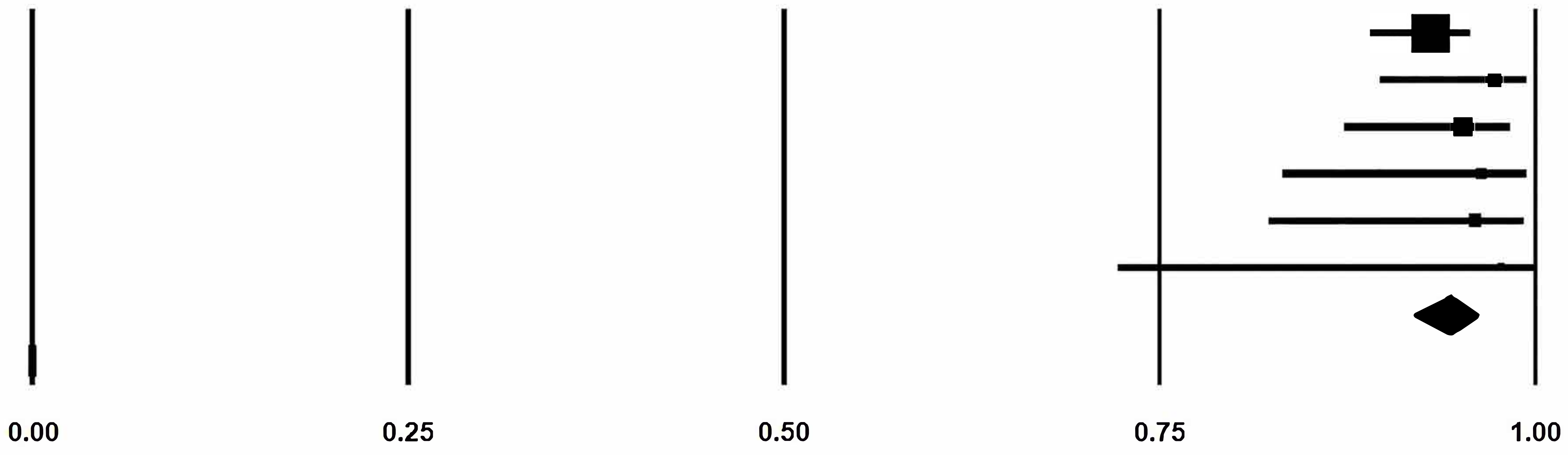

The I-squared statistic is 0% p0.702

Figure S15: 12-month Freedom from Reintervention

Study name	Event rate	Lower limit	Upper limit
Abdelhalim MA, et al.2023	0.895	0.850	0.928
Patrick RJ, et al. 2023	0.882	0.641	0.969
DiBartolomeo AD, et al. 2023	0.719	0.542	0.847
Yang G, et al. 2023	0.944	0.861	0.979
Marques de Marino P, et al. 2022	0.814	0.710	0.887
Benfor B, et al. 2022	0.670	0.487	0.813
Wang X, et al. 2022	0.949	0.817	0.987
Gallitto E, et al. 2022	0.710	0.546	0.833
Verzini F, et al. 2020	0.857	0.638	0.953
Pooled	0.839	0.759	0.896
Prediction Interval	0.839	0.518	0.962

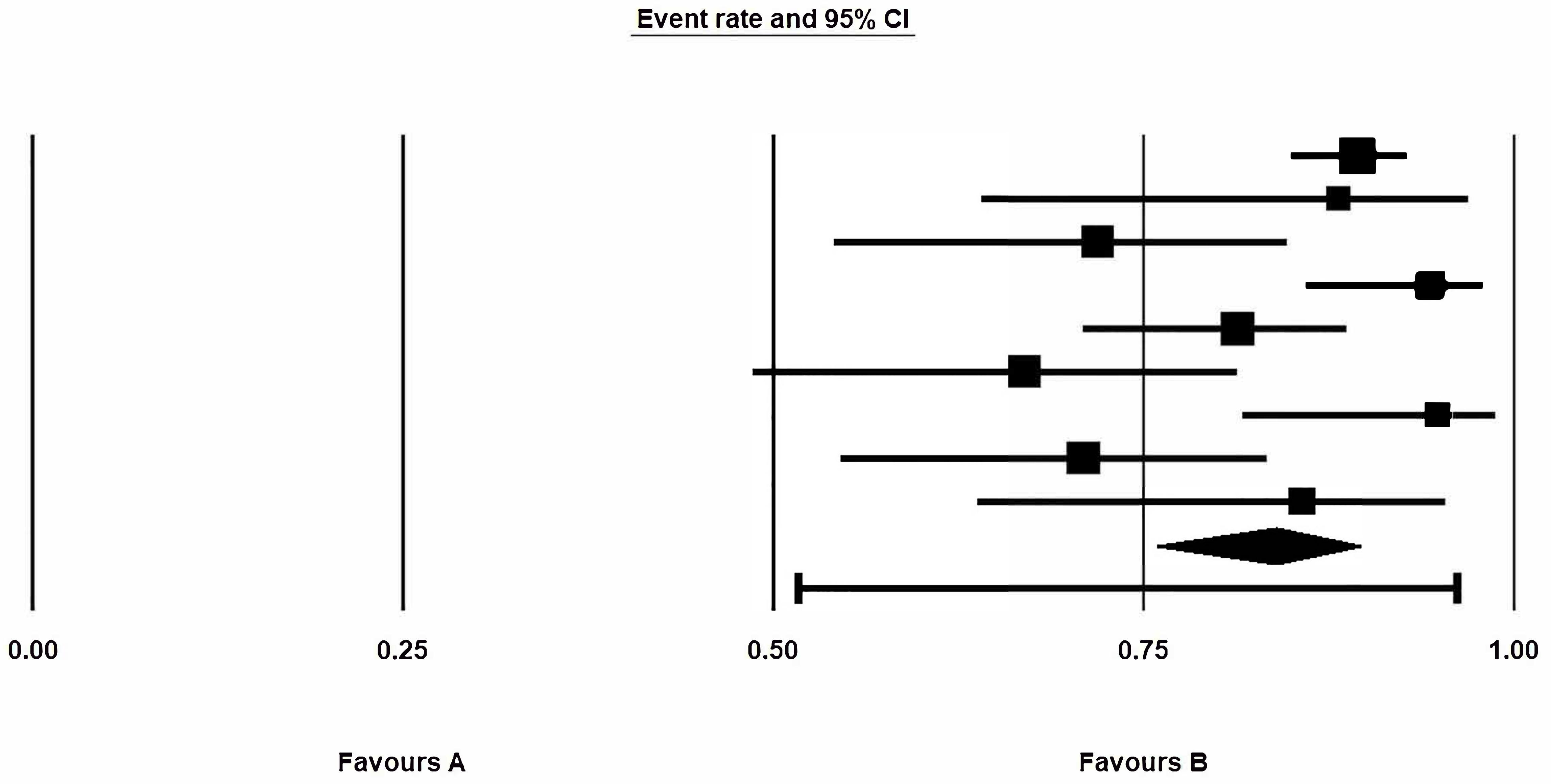

The I-squared statistic is 71% p0.000

Figure S16: 24-month Freedom from Reintervention

Study name	Event rate	Lower limit	Upper limit
Abdelhalim MA, et al.2023	0.869	0.821	0.906
Yang G, et al. 2023	0.902	0.809	0.952
Marques de Marino P, et al. 2022	0.569	0.455	0.676
Wang X, et al. 2022	0.949	0.817	0.987
Gallitto E, et al. 2022	0.710	0.546	0.833
Verzini F, et al. 2020	0.857	0.638	0.953
Pooled	0.828	0.687	0.914
Prediction Interval	0.828	0.245	0.986

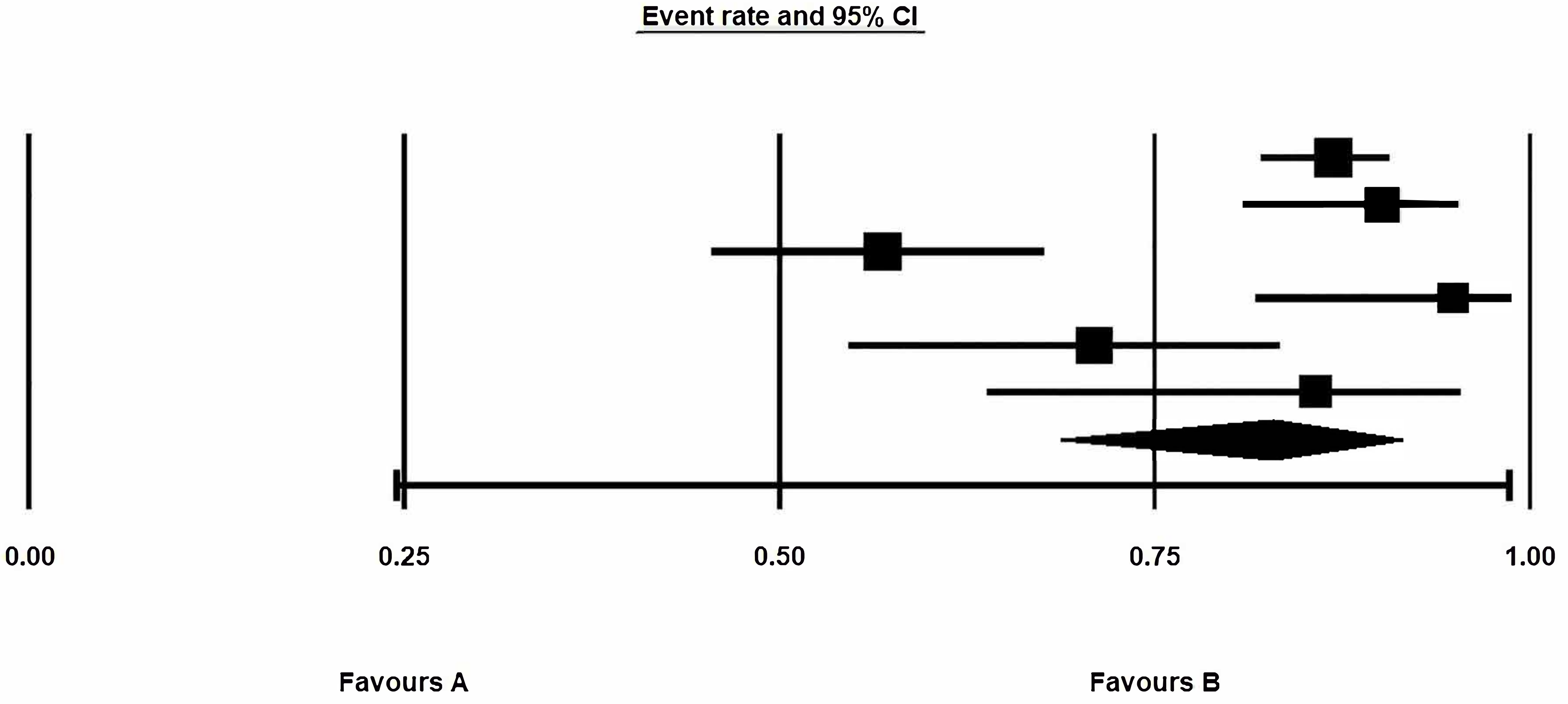

The I-squared statistic is 87.8% p0.000

Figure S17: 36-month Freedom from Reintervention

Study name	Event rate	Lower limit	Upper limit
Abdelhalim MA, et al.2023	0.831	0.779	0.873
Yang G, et al. 2023	0.858	0.757	0.921
Marques de Marino P, et al. 2022	0.539	0.426	0.648
Gallitto E, et al. 2022	0.660	0.496	0.793
Verzini F, et al. 2020	0.857	0.638	0.953
Pooled	0.761	0.606	0.868
Prediction Interval	0.761	0.182	0.979

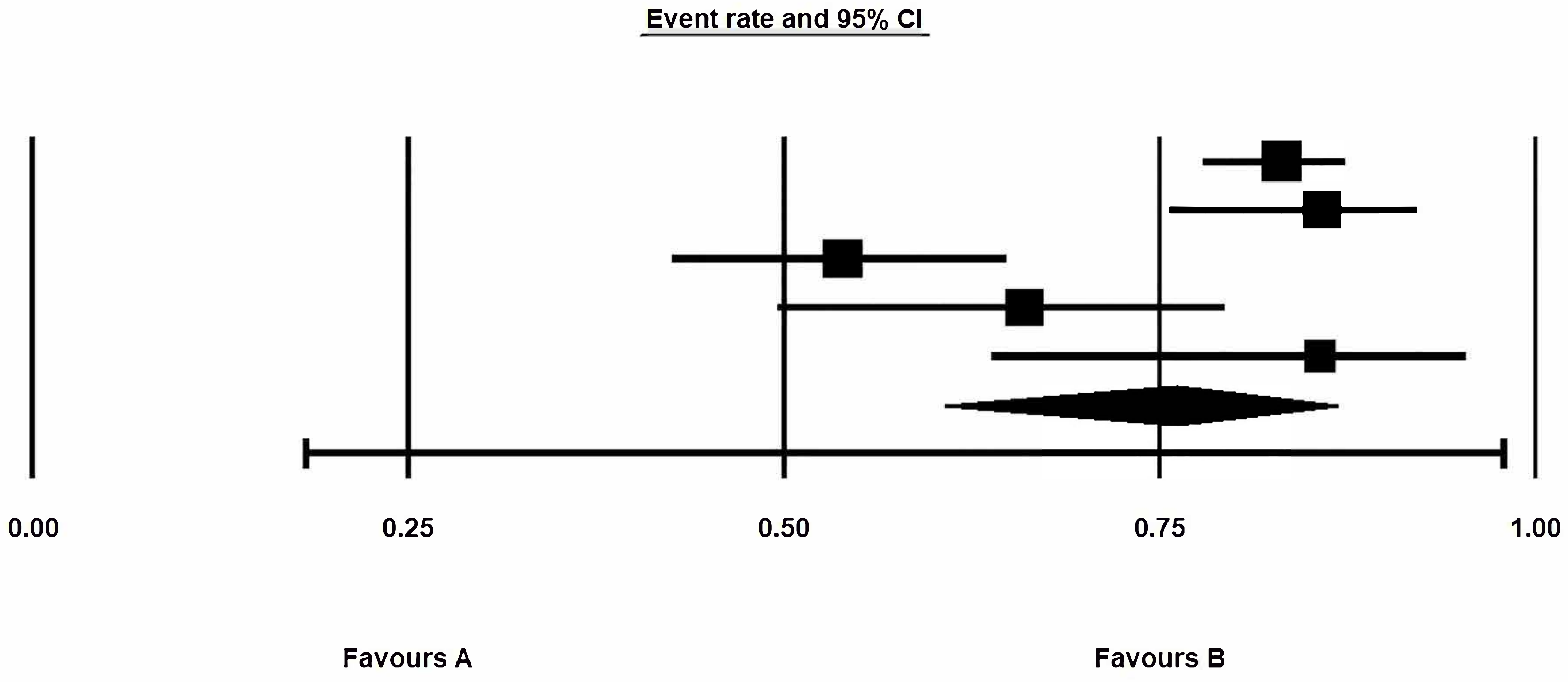

The I-squared statistic is 87.4% p0.000

Figure S18: Reinterventions IR per 100 person-years

Study name	Rate	Standard error	Variance	Lower limit	Upper limit	Z-Value	p-Value
	Statistics for each study						
Abdelhalim MA, et al.2023	0.262	0.023	0.001	0.217	0.307	11.358	0.000
Patrick RJ, et al. 2023	0.011	0.008	0.000	-0.004	0.027	1.414	0.157
DiBartolomeo AD, et al. 2023	0.410	0.114	0.013	0.187	0.632	3.606	0.000
Yang G, et al. 2023	0.043	0.013	0.000	0.016	0.069	3.162	0.002
Marques de Marino P, et al. 2022	0.171	0.031	0.001	0.111	0.231	5.568	0.000
Benfor B, et al. 2022	0.240	0.080	0.006	0.083	0.397	3.000	0.003
Wang X, et al. 2022	0.005	0.007	0.000	-0.009	0.020	0.707	0.480
Gallitto E, et al. 2022	0.122	0.035	0.001	0.053	0.190	3.464	0.001
Verzini F, et al. 2020	0.075	0.043	0.002	-0.010	0.159	1.732	0.083
Kitagawa A, et al. 2013	0.314	0.111	0.012	0.096	0.531	2.828	0.005
Pooled	0.126	0.027	0.001	0.073	0.179	4.647	0.000
Prediction Interval	0.126			-0.054	0.306		

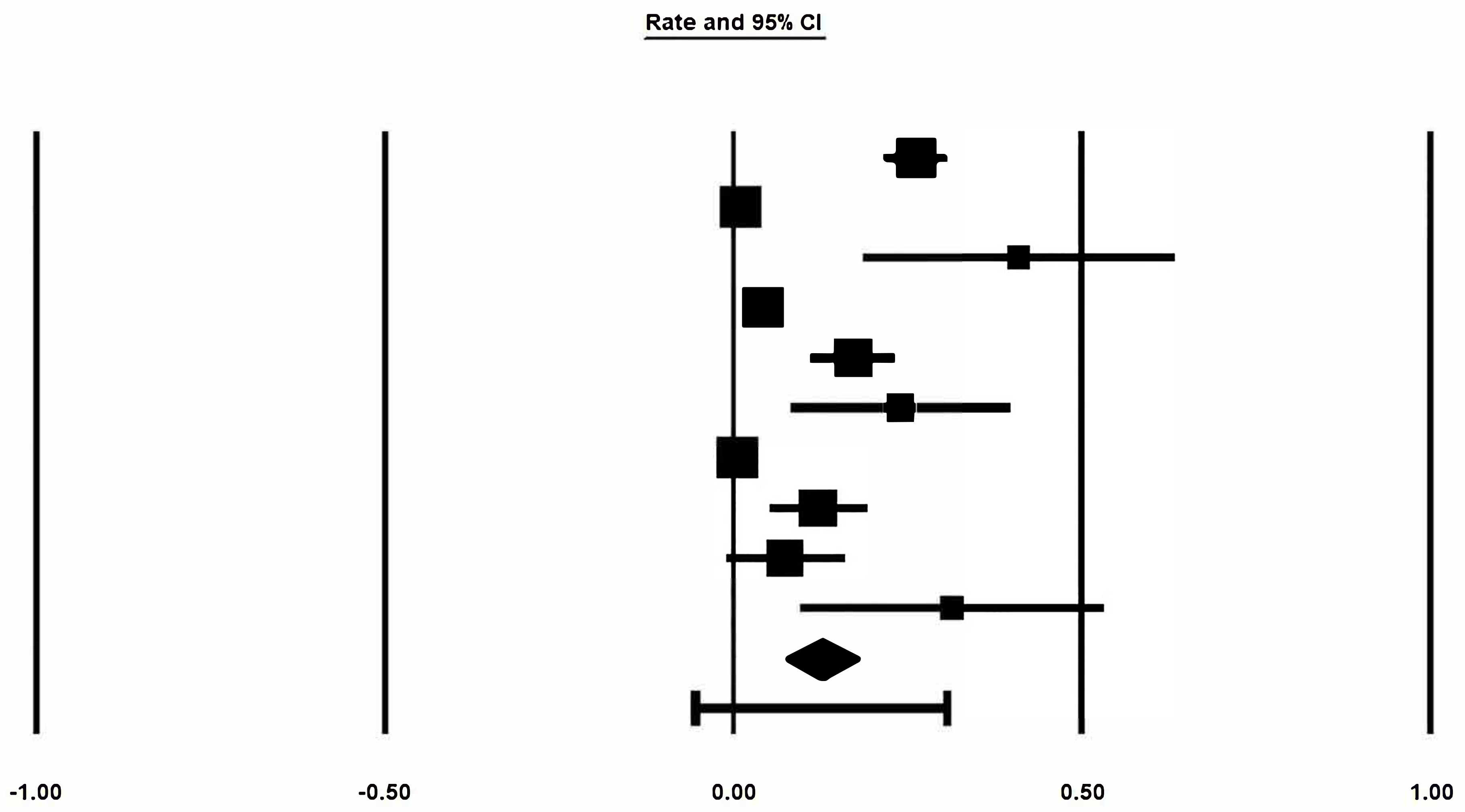

The I-squared statistic is 95% p0.000

Figure SI9: Type I/III Endoleak IR per 100 person-years

Study name	Rate	Standard error	Variance	Lower limit	Upper limit	Z-Value	p-Value
Abdelhalim MA, et al.2023	0.049	0.010	0.000	0.029	0.068	4.899	0.000
Patrick RJ, et al. 2023	0.006	0.006	0.000	-0.005	0.016	1.000	0.317
DiBartolomeo AD, et al. 2023	0.599	0.137	0.019	0.330	0.868	4.359	0.000
Yang G, et al. 2023	0.038	0.013	0.000	0.013	0.063	3.000	0.003
Benfor B, et al. 2022	0.293	0.088	0.008	0.120	0.467	3.317	0.001
Wang X, et al. 2022	0.021	0.015	0.000	-0.008	0.050	1.414	0.157
Gallitto E, et al. 2022	0.041	0.020	0.000	0.001	0.080	2.000	0.046
Verzini F, et al. 2020	0.075	0.043	0.002	-0.010	0.159	1.732	0.083
Kitagawa A, et al. 2013	0.392	0.124	0.015	0.149	0.635	3.162	0.002
Pooled	0.055	0.016	0.000	0.023	0.087	3.358	0.001
Prediction Interval	0.055			-0.041	0.151		

Rate and 95% CI

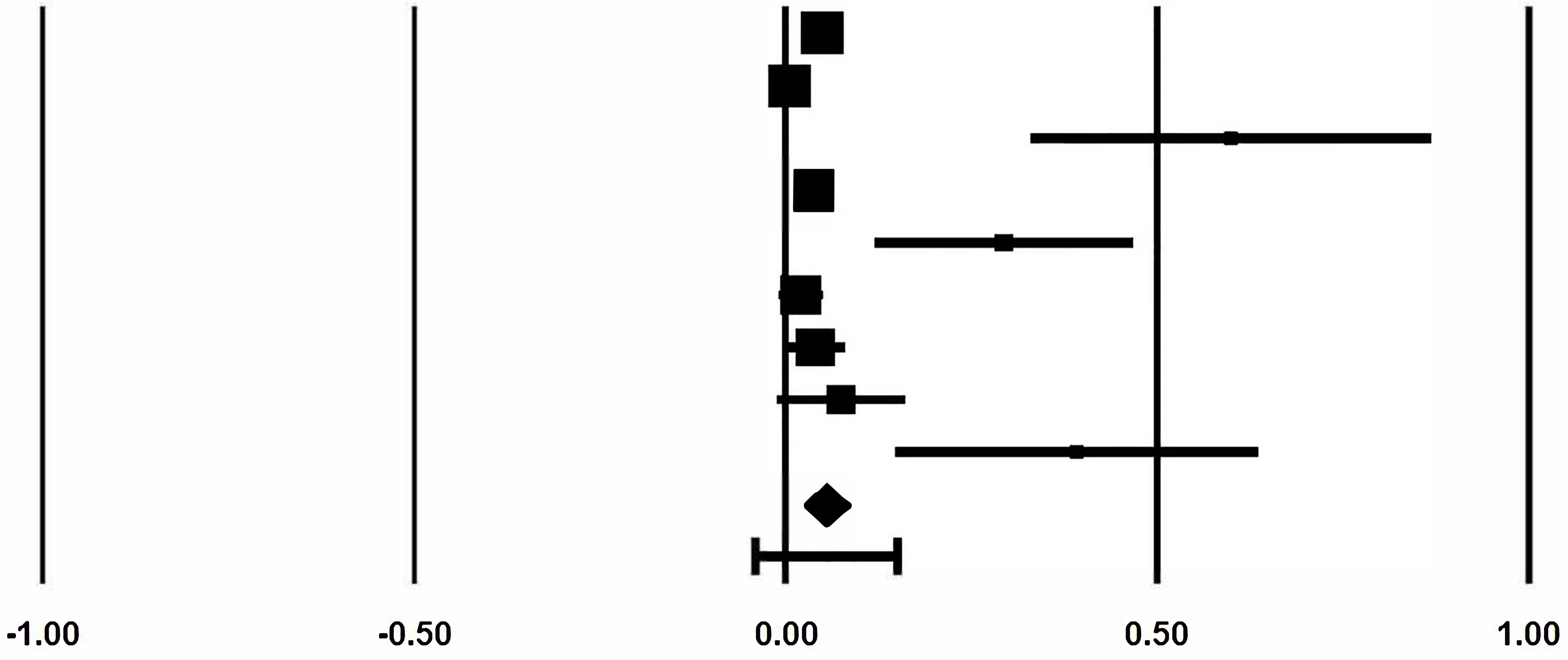

The I-squared statistic is 85.7% p0.000
